# Brain expansion in early hominins predicts carnivore extinctions in East Africa

**DOI:** 10.1111/ele.13451

**Published:** 2020-01-13

**Authors:** Søren Faurby, Daniele Silvestro, Lars Werdelin, Alexandre Antonelli

**Affiliations:** ^1^ Department of Biological and Environmental Sciences University of Gothenburg Box 461 SE 40530 Göteborg Sweden; ^2^ Gothenburg Global Biodiversity Centre Box 461 SE 40530 Göteborg Sweden; ^3^ Department of Computational Biology, Biophore University of Lausanne Lausanne Switzerland; ^4^ Swiss Institute of Bioinformatics Quartier Sorge 1015 Lausanne Switzerland; ^5^ Department of Palaeobiology Swedish Museum of Natural History Box 50007 SE 10405 Stockholm Sweden; ^6^ Royal Botanic Gardens Kew, Richmond Surrey TW9 3AE U.K

**Keywords:** anthropogenic, bayesian, carnivora, humans, pleistocene, pliocene, PyRate

## Abstract

While the anthropogenic impact on ecosystems today is evident, it remains unclear if the detrimental effect of hominins on co‐occurring biodiversity is a recent phenomenon or has also been the pattern for earlier hominin species. We test this using the East African carnivore fossil record. We analyse the diversity of carnivores over the last four million years and investigate whether any decline is related to an increase in hominin cognitive capacity, vegetation changes or climatic changes. We find that extinction rates in large carnivores correlate with increased hominin brain size and with vegetation changes, but not with precipitation or temperature changes. While temporal analyses cannot distinguish between the effects of vegetation changes and hominins, we show through spatial analyses of contemporary carnivores in Africa that only hominin causation is plausible. Our results suggest that substantial anthropogenic influence on biodiversity started millions of years earlier than currently assumed.

## Graphical abstract

While the anthropogenic impact on ecosystems today is evident, it remains unclear if the detrimental effect of the human lineage on co‐occurring biodiversity is a recent phenomenon or has also been the pattern for earlier hominin species. Focusing on fossil data from East Africa, the cradle of mankind, we find support for a strong negative effect on co‐occurring biodiversity caused by our ancestors already in the Pliocene. Our results suggest that substantial anthropogenic influence on biodiversity started millions of years earlier than currently assumed.

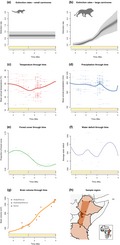

## Introduction

The far‐reaching impact of humans on natural ecosystems has prompted the proposal of a new geological epoch, the Anthropocene (Zalasiewicz *et al.* 2011). Yet, it remains unclear when human influence on biodiversity began, with most research focusing on the effects of *Homo sapiens* on late Pleistocene and early Holocene megafauna extinctions (Barnosky *et al.* 2004; Sandom *et al.* 2014). An earlier influence of hominins on biodiversity has, however, been proposed based on temporal changes in functional diversity in Africa (Werdelin & Lewis 2013a) and on the extinction of clades in areas inhabited by hominins but their survival elsewhere (Rhodin *et al.* 2015). Here, we provide the first explicit test of such early effects, focusing on East Africa. This may be the optimal region in which to investigate this question, given the rich representation of hominins in the fossil record and their long co‐existence with a diverse fauna.

The current diversity of large carnivores (i.e. species with a body mass greater than *c*. 21 kg; Carbone *et al.* 1999), is greater in East Africa than elsewhere in the world (Ripple *et al.* 2014), even though it is dwarfed by the diversity seen in Pleistocene systems in North America or Eurasia (Valkenburgh *et al.* 2016). The diversity in Africa was, however, much greater before the Pleistocene (Werdelin & Lewis 2005; Sandom *et al.* 2014). It was also substantially more variable and included the dogs, conical‐toothed cats and hyenas still present today, albeit with lower diversity, as well as extinct species of bears, saber‐toothed cats, and giant species of martens, otters and civets (Werdelin & Lewis 2005).

**Figure 1:**
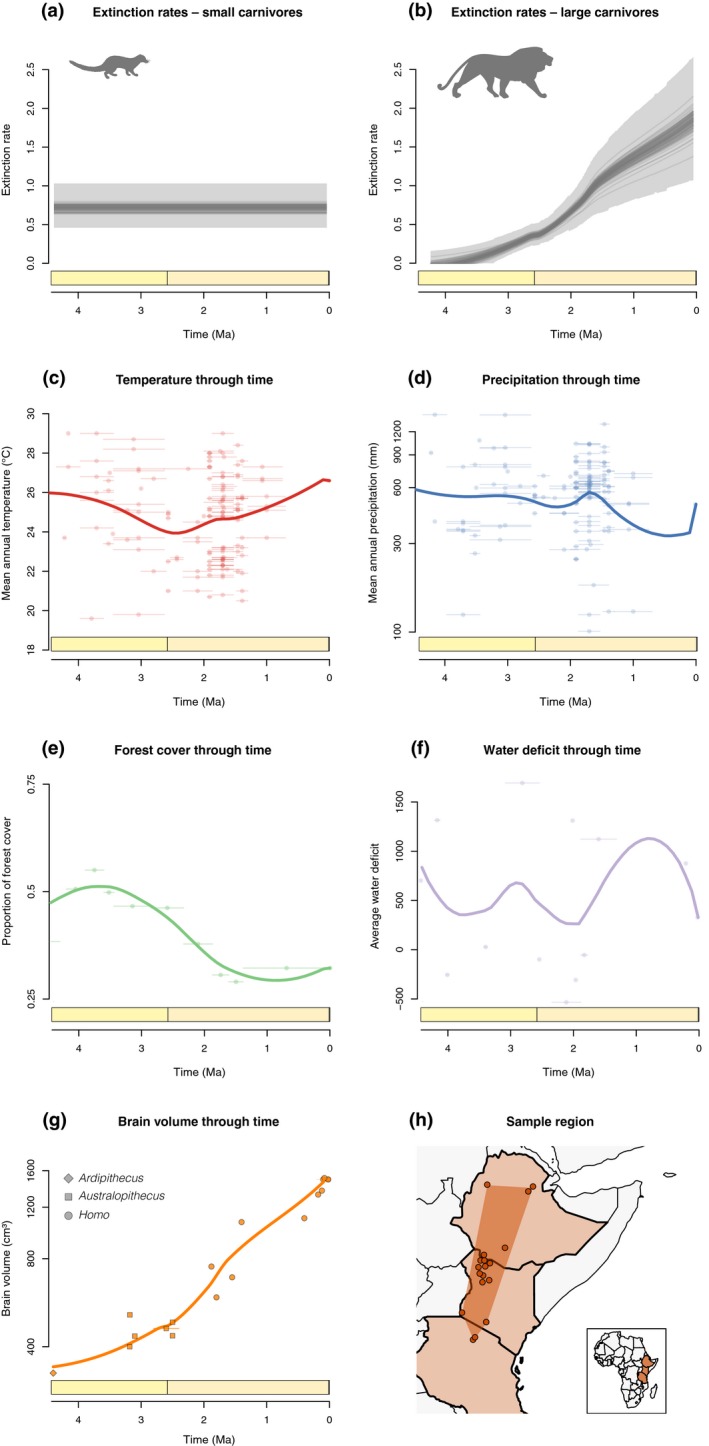
Potential drivers of carnivore extinctions in East Africa. (a and b) Extinction rates (expected number of extinction events per lineage per million years) through time, as inferred from the best‐fitting Bayesian model (Table S1) for small carnivores (i.e. a constant extinction rate model) and large carnivores (i.e. a model with linear correlation between extinction rates and brain volume). Dark lines show mean posterior rates inferred from 100 replicated analyses, where all fossils in each analysis are assigned a random age between the minimum and maximum age of the fossil site, and shaded areas indicate 95% credible intervals around rates. Horizontal bars above the time axis show the boundary between the Pliocene and the Pleistocene. (c–g) Potential predictors of the extinction patterns. (c) Mean annual temperature (Fortelius et al. 2016). (d) Mean annual precipitation (Fortelius et al. 2016). (e) Fraction of forest cover (Cerling et al. 2011). (f) Annual water deficit (Blumenthal et al. 2017). For (c–f) The raw data are depicted as circles with horizontal bars showing the dating uncertainties around each measurement. (g) Evolution of brain volume in hominins (White et al. 2009; Seymour et al. 2016). Diamonds, squares and circles indicate fossil measurements for species of *Ardipithecus*, *Australopithecus*, and *Homo*, respectively, with horizontal bars showing temporal uncertainties. In c–g, points were interpolated by computing the median of 1000 loess regressions obtained after resampling the dates of each measurement to incorporate temporal uncertainties. (h) Fossil sites. These analyses indicate that extinction rates in large carnivores (> 21 kg) are best explained by changes in hominin brain size or forest cover. In contrast, there is no evidence of rate variation in small carnivores, as would be expected in climate‐driven extinctions.

The simplest explanation for the decline of carnivore diversity in East Africa is climatic change. We know from contemporary data that herbivore diversity is strongly determined by climatic conditions, and that the diversity of carnivores is in turn tightly linked to the diversity of herbivores (Sandom *et al.* 2013). This link has been documented throughout the Neogene, with strong correlation between mammalian diversity and site productivity (Fritz *et al.* 2016). In East Africa, the climate has been relatively stable throughout the Neogene, although there is evidence for a small decrease in temperature and a moderate decline in annual precipitation from the late Miocene to the middle Pleistocene (Fortelius *et al.* 2016) and a moderate decrease in forest cover in the region, potentially linked to reductions in precipitation (Cerling *et al.* 2011) (Fig. 1c–f).

An alternative explanation for the decline in large carnivore diversity in East Africa is the effect of early hominins. This region, including the Turkana and Afar Basins, may be the birthplace of hominins and contains a well dated, continuous hominin presence for the last four million years (Myr) (Cerling *et al.* 2013), including many key fossils from all five hominin genera (*Homo*, *Australopithecus, Kenyanthropus, Paranthropus* and *Ardipithecus*). Importantly for our analysis, the carnivore fossil record from this period and region has been extensively studied (Werdelin & Lewis 2005, 2013a), making it a prime candidate for the assessment of early effects of hominins on biodiversity. Hominin brain size increased substantially from the late Miocene to the middle Pleistocene, from less than 500 cm^3^ to around 1500 cm^3^ (Seymour *et al.* 2016) (Fig. 1g, Table S1), and is temporally (perhaps causally) associated with the development of increasingly advanced behaviour and technology (Bramble & Lieberman 2004; Harmand et al. 2015; Semaw *et al.* 1997).

Hominins could potentially drive extinctions through at least three different mechanisms: direct hunting, scavenging or kleptoparasitism. Any one of them could lead to reduced nutrition being available to the carnivore guild. Direct hunting of herbivorous mammals is often discussed in later megafauna extinctions where high extinction is seen among herbivores (e.g. Ben‐Dor *et al.* 2011). Perhaps equally important for carnivores, however, could be a reduction in food quantity through hominin scavenging (i.e. usage of undefended carcasses) or kleptoparasitism (i.e. driving away carnivores or scavengers from defended carcasses before usage). The latter two processes are noteworthy in requiring less advanced behaviour and technologies than direct hunting. It has been suggested that early hominins used thorny branches to defend themselves against carnivores (Kortlandt 1980). Such branches would not be useful for hunting but may have been enough to force carnivores to give up a partially eaten carcass.

In order to test the importance of climatic versus hominin causation in driving carnivore extinctions, we developed a Bayesian analytical framework using the program PyRate (Silvestro *et al.* 2019). We analysed the large and small carnivore fossil records from East Africa, in conjunction with local proxies for climatic effects (annual temperature, annual precipitation, annual water deficit and forest cover) as well as hominin effects (brain size).

## Materials and methods

### Data

The dataset included 279 fossil occurrences and 88 species (Table S2), 9 of which are still present in the area, while the remaining 79 are extinct. The dataset was compiled by one of the authors (LW), primarily from first‐hand study and analysis of the fossil specimens. Carnivore species were split into ‘large’ and ‘small’ based on their body mass and the standard threshold of 21 kg (more details in *Supporting information*: *Fossil dataset*).

We retrieved information on evolutionary changes in hominin brain size (White *et al.* 2009; Seymour *et al.* 2016) and proxies for climate and vegetation. As potential climatic predictors, we used mean annual temperature (Fortelius *et al.* 2016), mean annual precipitation (Fortelius *et al.* 2016), percentage forest cover (Cerling *et al.* 2011) and annual water deficit (Blumenthal *et al.* 2017). All the environmental predictors are estimated based on the sites from which the carnivore fossils originated.

For both environmental predictors and brain size, we created temporal predictors by computing the median of 1000 loess regressions obtained after resampling the dates of each measurement to incorporate temporal uncertainties (see *Supporting information*: *Environmental and brain size predictors*).

### Extinction rate analyses

We performed the analyses of the fossil record using the Bayesian framework implemented in the program PyRate (Silvestro *et al.* 2019) to infer extinction and preservation rates through time in large and small carnivores (see *Supporting information*: *Fossil analysis*). We tested different extinction models, including non‐mechanistic null models where extinction rate can change over time through estimated rate shifts. We also tested mechanistic models in which extinction rates vary as a linear or exponential function of environmental predictors (palaeoclimate and palaeo–vegetation proxies). Lastly, we tested models with rate variation driven by changes in hominin mean brain size, to test for a causal link between hominin evolution and carnivore extinctions.

We quantified the fit of each model by estimating the respective marginal likelihoods and assessed the relative support using Bayes factors. To determine whether the brain size predictor is only significant after the average hominin brain reached a certain volume, we additionally implemented a new model where changes in brain size have non‐zero effects only after an estimated temporal threshold. To assess the support for mechanistic models, we compared their fit with that of null non‐mechanistic models in which changes in extinction rate are estimated from the data rather than testing specific *a priori* constraints (see *Supporting information*: *Model testing* and *Supporting information*: *Parameter estimation*). Thus, we emphasise that we are not only comparing climate, palaeo‐vegetation and brain size predictors against an unrealistically simple null model with constant extinction rate, but also against other time‐variable models with one or more rate shifts estimated from the data. This is important because apparent correlations may otherwise derive from the use of inadequate null models (see e.g. Rabosky & Goldberg 2015).

### Spatial analyses

In order to further assess the plausibility of each of the potential predictors in driving the observed changes, we analysed contemporary variation in the ratio of co‐occurring (non‐ pinniped) carnivores in continental Africa that are larger than 21 kg. These analyses were conducted on a Behrman projected grid with a column width of 1 degree giving areas of each cell of about 9000 km^2^. Data on distribution and body sizes were taken from Faurby et al. (2018) but originally come from multiple sources (Faurby & Svenning 2016; IUCN 2016; Smith *et al.* 2003).

We analysed the spatial data in a multiple correlation framework with log‐transformed annual precipitation (Worldclim v 1.4; Hijmans *et al.* 2005), mean annual temperature (Worldclim v 1.4; Hijmans *et al.* 2005), forest cover (Hansen et al. 2013) and human footprint (Venter *et al.* 2016) as potential predictors of the local fraction of large carnivores (those with a body mass > 21 kg). We acknowledge that high human impact could be associated with increased deforestation, but the current data do not allow a separation of natural versus hominin‐induced effects on forest cover. We did not include water deficit in our spatial analyses because it is already derived from annual temperature and annual precipitation. We note here that since the fossil analyses described above are all univariate, correlations between potential predictors are not a problem for them, which is why we were able to include water deficit in the temporal but not the spatial analyses. Regressions were conducted both through classical linear regression and through simultaneous autoregressive models with spatial error (i.e. SAR^err^ models). We tested 40 different neighbourhoods (10 with a fixed number of neighbours between one and ten, and 30 containing all combinations of cells up to 250, 500, 750, 1000, 1250 or 1500 kilometres away with either of the five default weighting schemes). The best neighbourhood (the four closest cells) was chosen as the one minimising the Akaike Information Criterion (AIC). Both the linear regression and the SAR^err^ regressions were conducted in R 3.4.1 (R Core Team 2017) using glm (R Core Team 2017) and errorsarlm from the package spdep (Bivand & Piras 2015).

We extrapolated the contemporary pattern to evaluate whether any temporal changes in the fraction of large carnivores could be expected to occur based on climatic changes. For this, we fitted a line based on the coefficients from the linear regression model and the palaeo–environmental reconstructions (i.e. the outputs of the loess regressions for mean annual temperature, mean annual precipitation and estimated forest cover). Since we were solely interested in understanding changes in the fraction of large carnivores rather than absolute values, we shifted the line so the predicted value at 4 Ma was the same as the median across all PyRate runs.

### Inter‐continental comparison

The reduction in forest cover over the last few million years is a global phenomenon (Osborne 2008). If a reduction in forest cover in Africa caused a decline in large carnivores, we should therefore expect a similar decline elsewhere, including North America. As a means to further validate our analyses, we inferred changes in carnivore fossil diversity in North America for the same time period as for the East African analysis. For this, we followed the taxonomy of Faurby *et al. *(2019) and used the records they gathered. We scored presence or absence of each species by geological age based on the midpoint of the minimum and maximum age of the fossils and range‐through lifespans (i.e. if a species is known from fossils in both the preceeding and the following geological age, its presence in the intermediate age is also assumed even if there are no fossils of the species within it). We then scored the size of all North American carnivores using the same threshold as in the African record (21 kg; Carbone *et al.* 1999) and calculated the fraction of large carnivores for each geological stage following Gradstein *et al *(2012).

## Results

### Analytical support for hominin causation

**Figure 2:**
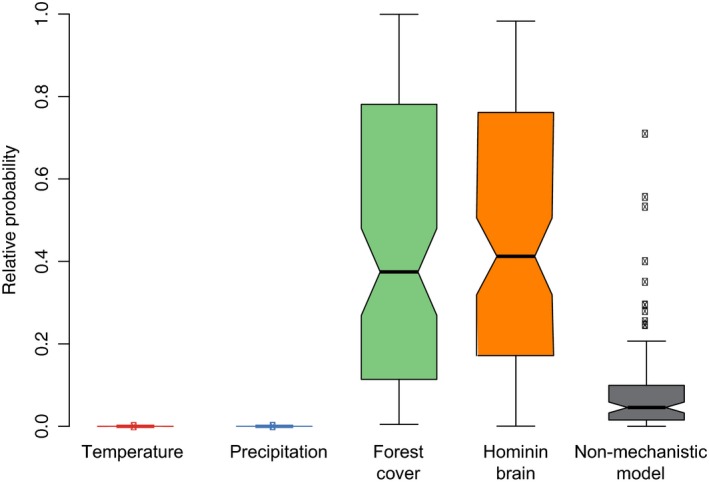
Relative probabilities of alternative extinction models. Extinction rate was modelled as a function of local proxies for mean annual temperature, mean annual precipitation, forest cover, water deficit and hominin brain volume. The fit of these models was compared with that of a non‐mechanistic null model in which rate changes through time were modelled by estimated shifts, i.e. without any explicit underlying hypothesis about causes of rate variation. Of the 15 models tested in total (Table S3), we here show the best fitting ones for each of the five main categories: extinction models with linear correlation for temperature and precipitation, exponential relationship for forest cover, linear correlation without thresholds for hominin brain, and one rate shift for the non‐mechanistic models. We computed the relative probabilities by normalising model log marginal likelihoods. The boxplots show the range of relative probabilities obtained from 100 replicated analyses (see Methods). The limit of the boxes is the upper and lower quartiles, with the median shown with a thick line. The notches extend to 1.58 times the interquartile range divided by the square root of sample size (*N* = 100). Whiskers extend to 1.5 times the inter‐quartile‐range and any outliers outside this are shown as circles.

Our analysis recovered no support for any temporal change in the rate of extinction of small carnivores; in contrast, we found a sharp increase through time in the extinction rate of large carnivores (>21 kg) (Fig. 1; Table S3). Among the variables surveyed (Fig. 1), we found significant support for two scenarios for this increase in extinction rate among large carnivores: either an anthropogenic impact, indicated by a correlation between extinction rate and hominin brain size; or, alternatively, an increased extinction rate driven by reduced forest cover (Fig. 2). On the other hand, we found no support for the effects of temperature, precipitation or water deficit (Fig. 2; Table S3). Taken together, models where extinction rates were driven by changes in average hominin brain size or vegetation cover not only substantially outperformed temperature/precipitation‐driven predictions (log Bayes factors > 10), but also outperformed other non‐mechanistic models with time‐variable extinction rates (Fig. 2) (log Bayes factors 4.38–6.1; Table S3; Fig. 1). Vegetation cover and brain size are, however, tightly correlated temporally (*R* ^2^ = 0.87) and we cannot identify the cause solely based on the analysis of the fossil record.

**Figure 3:**
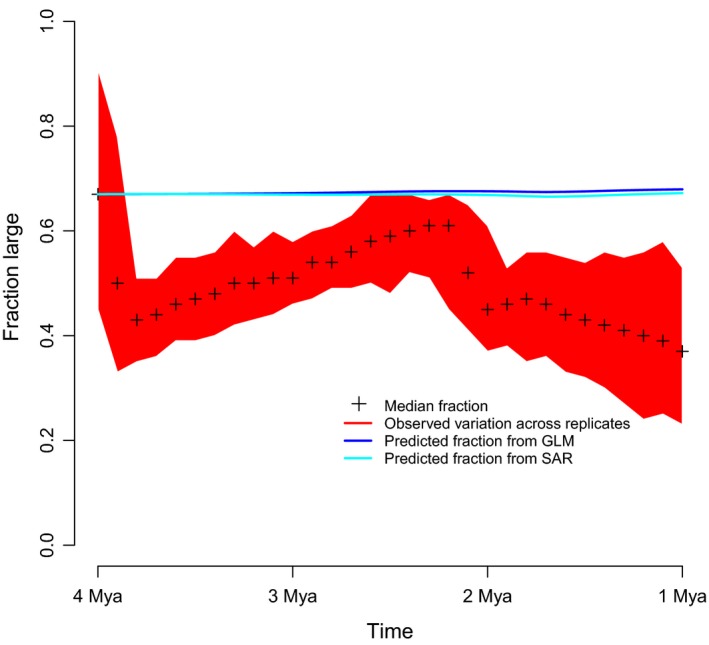
Predicted and observed temporal changes in the fraction of large carnivores (those with a body mass >21 kg). Median values across all 100 replicates are given, along with the full range across replicates. We also show the median predicted change, based on the climatic conditions outlined in Table 1. In order to focus on the predicted temporal change rather than the absolute predictions, we transformed the predictions to have the empirical slope but the same value at 4 Ma as the median from the fossil analyses.

To tease apart the effects of vegetation cover and hominin brain size on carnivore extinction, we supplemented our temporal analyses with spatial analyses of contemporary distribution patterns. These analyses allowed us to assess the plausibility that the observed climatic and vegetation changes in the region drove changes in carnivore diversity. We focused on the large carnivore fraction of total carnivore diversity, since our temporal analyses showed a constant extinction rate for small carnivores but a steadily increasing rate for large carnivores, which should create substantial temporal change in this ratio (Fig. 1). For contemporary faunas, we found that this fraction was negatively affected by the degree of human influence on the area and by increased precipitation, but effectively independent of temperature. Forest cover was also potentially moderately important, but inconsistent between regression methods (Table 1). We predicted the expected changes in the fraction of large carnivores in the past based on measured changes in climate and vegetation, and on the correlation coefficients estimated from the spatial analysis (Fig. 3). The results indicated that the fraction of large carnivores should have remained nearly constant without anthropogenic influences, in stark contrast to the observed pattern of steady decline (Fig. 3). This discrepancy supported the expectations from a steady increase in the anthropogenic footprint in the area over time. In summary, although the contemporary analysis suggested that climatic drivers (especially precipitation) may be important for carnivore community composition, there is no indication that Pliocene–Pleistocene climatic changes could have caused the observed changes.

**Table 1:** Predictors of fraction of large carnivores

	GLM			SAR^err^		
	Estimate	Spatial range	Temporal range	Estimate	Contemporary range	Temporal range
Intercept	0.000 (0.014)	0.276		0.005 (0.073)	0.242	
Precipitation	−0.314 (0.023)	−0.106 to 0.000	−0.081 to −0.074	−0.435 (0.058)	−0.147 to 0.000	−0.112 to −0.102
Temperature	−0.047 (0.015)	−0.031 to −0.011	−0.027 to −0.025	0.020 (0.032)	0.005 to 0.013	0.010 to 0.012
Forest cover	−0.0614 (0.0191)	0.000	0.000	0.065 (0.028)	0.000 to 0.002	0.001 to 0.001
Human footprint	−0.292 (0.019)	−0.192 to 0.000		−0.094 (0.017)	−0.061 to 0.000	
Pseudo *R* ^2^	0.307			0.284		
AIC	8213			3619		

The environmental effect on the fraction of large mammals of: precipitation (log10 transformed annual precipitation in millimetres), temperature (annual temperature in degrees Celsius), and human footprint; based on either a standard linear regression or a spatial SAR^err^ regression. The first columns for GLM and SAR^err^ provide point estimates and standard errors based on regressions with both the dependent and independent variables standardised to have a mean of 0 and a standard deviation of 1. In addition to the effect size, we provide the product of the estimated effect size and the range in the predictor in contemporary Africa (i.e. the spatial range), and the product of the effect size and the minimum and maximum value for the climatic predictors estimated within the temporal study period (temporal range). For SAR^err^, we estimate the Pseudo *R* ^2^ only based on the effect sizes and not the spatial parts of the predictions.

While we detected a drastic decline in the fraction of large carnivores in Africa, there was no indication of a similar decline in North America. The fraction of large North American carnivores was almost constant, or perhaps slightly increasing through time. The fractions in the Pliocene and latest Miocene were 34–37%, slightly increasing to 42–43% in the two stages in the Pleistocene (Table S4). Because changes in forest cover did not have an effect on the diversity of North American carnivores, this result further weakens the case that a decline of large carnivores in Africa could be attributable to changes in forest cover.

### Potential mechanisms behind the hominin effect

The analyses described thus far show support for a hominin causation of carnivore extinctions but cannot distinguish whether they were triggered by direct hunting of herbivores, by scavenging and/or by kleptoparasitism. The relative importance of direct hunting can potentially be estimated by temporal analyses: the latter two mechanisms are plausible for the entire 4‐million‐year time period considered, whereas direct hunting would require tool use and/or long‐distance pursuit, which did not appear until at a later stage (Bramble & Lieberman 2004; Harmand et al. 2015; Semaw *et al.* 1997). To tease apart these possibilities, we tested additional models where the relationship between extinction rate and brain size only started after an estimated threshold time, to reflect the development of key innovations, such as those necessary for efficient direct hunting. In this model, the extinction rate is constant until a threshold time, after which it is a function of a predictor (in this case brain size), with the threshold time being estimated from the data.

Hominins are thought to have started using stone tools about 3.3 million years ago (Ma) (Harmand et al. 2015) and, by 2.6 Ma (the Oldowan industry), tool‐use was widespread (Semaw *et al.* 1997). Physiological and mechanistic adaptations to efficient long‐distance movement and potential endurance hunting are suggested to have been in place by around 2.0 Ma (Bramble & Lieberman 2004). We found no evidence that the anthropogenic effects on extinction would have started later than 4 Ma: models with an estimated threshold time were rejected against the model where the relationship between brain size and extinction persisted for the entire period (log Bayes factors = 2.29–6.25; Table S3).

## Discussion

Our results provide strong support for a hominin causation of the increases in extinction rate among large carnivores in East Africa in the Pliocene and Pleistocene. Our results further point towards scavenging and kleptoparasitism as driving the initial extinction pattern. Later, increased brain size and novel locomotor adaptations in hominins likely led to increased levels of active hunting of herbivores and therefore reduced prey availability for the carnivores. Extinction rates further increased with the progressive invention of more advanced tools (Režek *et al.* 2018) and the evolution of adaptations promoting long distance movement. Our results thus contribute to the increasing recognition of a more prolonged and multi‐faceted hominin exploitation of large mammal resources than traditionally assumed (Thompson *et al.* 2019).

If the extinction of East African carnivores was primarily driven by hominins rather than by climatic changes as we propose, we should expect a difference between the extinction patterns for large and small carnivores, similar to the pattern seen in the spatial analysis of contemporary patterns (Table 1). Carnivores smaller than *c*. 21 kg generally feed on a large number of smaller prey items, with short handling time for each prey item. In contrast, large carnivores feed through rare successful hunts on larger prey items (Carbone *et al.* 1999). The effect of human kleptoparasitism should therefore be negligible for smaller carnivores but detrimental for larger carnivores. Our results show no evidence for any increase in extinction rates for small carnivores through the last 4 Myr: the simplest model with constant extinction rate is not rejected against the alternatives (log Bayes factors < 2, Table S3). These results further reject the models of climatically driven extinction, which predict that small and large carnivores should be affected similarly by climatic changes (Fritz *et al.* 2016). We also note that the temporal variation for all climatic variables (Fig. 1) is well within the range of contemporary climate data from elsewhere in Africa, whereas the fossil diversity of large carnivores is completely outside of the spatial variation observed today.

Kleptoparasitism whereby humans steal food from lions has been repeatedly observed in contemporary Africa (Schoe *et al.* 2009). There is also direct evidence for competitive interactions between hominins and giant hyenas (*Pachycrocuta brevirostris*) on the same carcass from the Pleistocene in Europe (Espigares *et al.* 2013). It is therefore plausible that kleptoparasitism was also important for earlier hominins. Hominins may be too fundamentally different from other animals for direct comparisons, but we note that the rate of kleptoparasitism in birds is known to be associated with cognitive ability (Morand‐Ferron *et al.* 2007), which would further support the interpretations of this study.

We found only moderate support (BF = 2.29) for a model without a threshold time, and this provides weak evidence against a model of large carnivore decline driven by direct hunting. This relatively weak support does not imply a weak inference of a human causation of the extinction pattern, however. A similar increase in the extinction of large carnivores, with no equivalent extinction of small taxa, should be observed if hominins competed directly through hunting, rather than through kleptoparasitism. This is because hunting hominins are likely to behave in similar ways to other carnivores (Carbone *et al.* 1999) by focussing on relatively large prey (i.e. species of approximately the same body mass as themselves), selecting a few large prey items, rather than many small, and therefore competing more with large than with small carnivores.

Discussions of potential interactions between hominins and individual species or species groups are necessarily speculative, but the available data strongly suggest a pattern where hominins progressively outcompeted particular functional groups while having limited effects on others. A recent paper discussing a sharp decline in East African megaherbivores from the Miocene to the Pliocene presents an alternative view of the extinction of a particular functional group, the saber‐toothed cats (Faith *et al.* 2018). The saber‐toothed cats have historically been interpreted as relying more on megaherbivores than any other carnivores, so if any group should suffer from a decline in megaherbivores it should arguably be them. Recently, however, stable isotopes and functional morphology have shown that saber‐toothed cats were not megaherbivore specialists but rather relied on more moderate‐sized herbivores like all other carnivores (Andersson *et al.* 2011; Bocherens 2015). Since the morphologically specialized saber‐toothed cats, which have been suggested to be megaherbivore specialists, behaved similarly to other carnivores, we consider it highly unlikely that any carnivores specialised on megaherbivores within our time period. We therefore also consider it unlikely that a shift in the size distribution of herbivores should cause the extinction patterns reported here. We further discuss changes in the diversity of the individual carnivore groups in *Supplementary information* *: Pattern by sub‐guild.* In that discussion, we argue that a climatic causation for the extinctions is once again unlikely when diversity is investigated in smaller subgroups.

We stress that our hypothesis does not require hominins to consume a large proportion of meat in their diet, at least not for the initial part of the extinction process. Our analyses rather support a scenario where increasingly cognitively advanced hominins were able to exploit a wider array of food items as a function of more advanced behaviour and eventually more advanced tool use. As hominins were able to exploit additional niche elements, their density could increase and therefore the combined meat intake of hominins may have been substantial, even if meat was only a moderate constituent of their diet. This argument is similar to observations on the ecological effect of omnivores in contemporary ecosystems. Omnivores generally occur at substantially higher population densities than strict carnivores (Pedersen *et al.* 2017). Consequently, species like the American black bear are in some regions the most important mammalian predators, even though the vast majority of their caloric intake is plant‐based (Vreeland *et al.* 2004).

An increase in their total meat consumption, at least initially, could have been driven by increased population size. Hominin populations could potentially have increased due to increased cognitive abilities and tool use (Kortlandt 1980), thus reducing death rates of hominins by top predators. It is also likely that pre‐hominin carnivore communities had low resilience to increased competition. Evidence from tooth breakage among Late Pleistocene carnivores from North America suggests that there was intense competition for prey in pre‐hominin ecosystems (Ripple & Van Valkenburgh 2010). Even a relatively moderate decline in available food –whether from prey stealing and scavenging by increasingly intelligent hominins, or from lower prey abundance due to direct hunting by tool‐using hominins – could therefore generate the substantial increase in extinction rate among large carnivores documented here (Fig 1).

Our results indicate that humans have been substantially modifying ecosystems from the time of their origin, and that this influence had already begun among earlier hominin lineages. Humans have been considered a hyperkeystone species (Worm & Paine 2016) due to their unique ability to completely transform ecosystems. Our results here suggest that this unique designation could be extended to earlier *Homo* species or even other hominins.

## Funding

Funding for this work was provided through a Wallenberg Academy Fellowship from the Knut and Alice Wallenberg Foundation, by the Swedish Research Council (B0569601, 2015‐04748, and 2017‐03862), the European Research Council under the European Union’s Seventh Framework Programme (FP/2007‐2013, ERC Grant Agreement n. 331024) and the Swedish Foundation for Strategic Research. Funding for work on African carnivores by LW has been provided by a series of grants from the Swedish Research Council.

## Competing interests

Authors declare no competing interests.

## Statement of authorship

SF, DS, LW and AA designed the study. LW validated the taxonomy for African carnivores. DS and SF analysed the data. SF and DS led the writing of the paper with input from LW and AA. All authors approved the submitted version.

## Data Availability

All data gathered for this study are available in the supplementary tables which are also uploaded to Zenodo (https://doi.org/10.5281/zenodo.3521578). All codes are available on GitHub (https://github.com/dsilvestro/PyRate). The scripts used for running individual analyses are available from the authors upon request.
