# Supplementary material for: Brain expansion in early hominins predicts carnivore extinctions in East Africa
Source: Ecol Lett. 2020 Jan 13;23(3):537–44. doi: 10.1111/ele.13451 (PMC7079157; doi:10.1111/ele.13451)
Supplement: Supplementary file 1 [file ELE-23-537-s001.docx]

**Supplementary information for:
*Brain expansion in early hominins predicts carnivore extinctions in East Africa***

**Authors:** S. Faurby^1,2,*,†^, D. Silvestro^1,2,3,4,†^, L. Werdelin^5^, A. Antonelli^1,2,6^

**Affiliations:**

^1^Department of Biological and Environmental Sciences, University of Gothenburg, Box 461, SE 40530, Göteborg, Sweden

^2^Gothenburg Global Biodiversity Centre, Box 461, SE 40530 Göteborg, Sweden

^3^Department of Computational Biology, Biophore, University of Lausanne, Lausanne, Switzerland

^4^Swiss Institute of Bioinformatics, Quartier Sorge, 1015 Lausanne, Switzerland

^5^Swedish Museum of Natural History, Department of Palaeobiology, Box 50007, SE 10405 Stockholm, Sweden

^6^Royal Botanic Gardens, Kew, Richmond, Surrey, TW9 3AE, U.K.

*Correspondence to: Soren.Faurby@bioenv.gu.se

^†^ Both authors contributed equally

**Supplementary Materials and Methods**

Fossil dataset

The dataset included 279 fossil occurrences and 88 species (Table S2), 9 of which are still present in the area, while the remaining 79 are extinct. The dataset was compiled by one of the authors (LW), primarily from first-hand study and analysis of the fossil specimens. Much but not all of this material has been published (South Turkwel, Kenya (Werdelin 2000); Kanapoi, Kenya (Werdelin 2003; Werdelin & Manthi 2012), Laetoli, Tanzania (Werdelin & Dehghani 2011); Woranso-Mille, Ethiopia (Werdelin et al. 2013); Koobi Fora, Kenya (Werdelin & Lewis 2013b)). A few faunal lists were obtained from published literature and critically evaluated (e.g. Omo, Ethiopia (Howell & Petter 1976); Olduvai, Tanzania (Petter 1973)). When studying fossils where genetic analyses are impossible, perfect identifications cannot be guaranteed and species designations can occasionally vary between experts for some specimens. We stress, however, that since all taxonomic decisions for this paper were conducted by the same taxonomist the data should have a very strong internal consistency. They should therefore be relatively safe from issues of taxonomic inconsistency when different groups are treated by taxonomists with overall tendency to either split or lump taxa which easily can generate spurious conclusions in macroevolution (Faurby et al. 2016).

We categorised carnivore species based on body mass using a threshold of 21 kg. This value corresponds to the well-known split among extant carnivores (Carbone et al. 1999) to distinguish between prey exploitation patterns in large and small taxa (36 and 52 species, respectively). The dataset spanned the past ca. 4.3 Myr, with most of the occurrences deriving from the period 4–1 Ma. Most fossil occurrences were dated based on the boundaries of their stratigraphic ranges and therefore assigned a range of ages, rather than a point estimate. The median size of age ranges across all occurrences in our dataset was very small, approximately 0.22 Myr, indicating high precision in the underlying data. We incorporated these age ranges as dating uncertainties in the subsequent analyses (see below), by generating 100 randomized datasets in which the age of each occurrence was a random uniform sample from its age range. Thus, all the analyses described below and in the main text are a summary of 100 replicated analyses. The extinction rates inferred from the different datasets were largely consistent across replicates (relative errors ranging between 0.1 and 0.19).

Climatic and brain size data

We retrieved information on evolutionary changes in brain size (volume in cubic centimetres) among hominins from published sources (Seymour et al. 2016; White et al. 2009). We only considered measurements of specimens from Africa, excluding *Homo naledi* as a clear outlier unrelated to the main hominin evolutionary line (Dirks et al. 2017). Our final dataset comprised 22 measurements of brain size spanning 4.4 to 0 Ma (Table S2). We obtained four previously estimated proxies for local palaeo-environment. Two of these, mean annual temperature and mean annual precipitation, were estimated based on the tooth morphology of the herbivore communities in the fossil sites (Fortelius et al. 2016). The third, water deficit, was estimated based on stable oxygen isotopes in fossil herbivore teeth (Blumenthal et al. 2017). The fourth, forest cover, was estimated based on stable carbon isotopes in fossil soils (Cerling et al. 2011), relying on the results from the Omo-Turkana Basin. To obtain time-continuous variables we interpolated available measurements for each parameter (hominin brain size, mean annual temperature and precipitation, mean forest cover) using loess regressions in R 3.3.3 (R Core Team 2017), with default settings. We log10-transformed mean annual precipitation and hominin brain size prior to all analyses outlined below. Each measurement was assigned a time range reflecting dating uncertainties. We accommodated these uncertainties by running the loess regression function 1,000 times, after resampling the ages of the measurements from uniform distributions matching the respective temporal ranges. Finally, we calculated the median of the 1,000 regression lines and used it as proxy for hominin brain size and local climatic conditions.

This methodology treats all variation in climatic conditions as measurement error rather than spatial variation in the underlying data. This is not entirely correct, since there is spatial variation in the climate in the region (Fortelius et al. 2016). If the climatic variation is largely spatial rather than temporal it should, however, drive spatial rather than temporal variation in diversity, making it an unlikely predictor for temporal changes in the first place. The climatic predictors for precipitation, temperature and aridity are furthermore based on the herbivore fauna of the very same fossil sites for which we analysed the carnivore diversity. If underlying spatial variation led us to underestimate temporal variation, the same problems should be present in both the carnivore diversity we analysed and the climatic predictors.

We used brain size as a measure of hominin cognitive ability, but we acknowledge that intelligence is highly complex. Support for brain size/intelligence relationships generally relies on relative brain size (Benson-Amram et al. 2016). In the fossil record, brain size and body size coevolved and hominins progressively became larger and bigger brained (Seymour et al. 2016). This could mean that the effects we discuss here may partly be due to hominins becoming physically stronger rather than smart enough to be efficient hunters/ scavengers/ kleptoparasites. However, supporting our interpretation of increased cognitive ability, we note that blood flow to hominin brains increased faster than brain size in hominin evolution, which suggests higher energetic investment in brain function producing sharper increases in cognitive abilities than expected just by size changes (Seymour et al. 2016).

The data we used for brain-size measurements focus on the genera *Australopithecus* and *Homo,* for which the use of tools is confirmed. These hominins co-existed with *Paranthropus* and it has been proposed that this genus could also have been a tool-maker, a possibility that remains debated (Wood & Strait, 2004). *Paranthropus* is often considered a specialist plant-eater, while *Homo* species may be more adaptable in their diet, although there is substantial uncertainty about the diet of both genera (Wood & Strait, 2004). The scenario we tested matches the normal understanding of the ecology of *Homo*, but since both genera appear relatively frequently in the region we cannot discriminate between the effects of those two genera in our analyses.

Fossil analysis

We analysed fossil occurrence data using the program PyRate, which requires a species-level taxonomy but not an underlying phylogeny (Silvestro et al. 2019). PyRate implements Bayesian algorithms that combine models of preservation (fossilization and sampling) and birth-death stochastic processes to estimate the lifespans of lineages and infer the origination and extinction rates through time. We used this analytical framework to infer lineage origination and extinction times, while taking into account incomplete sampling and varying preservation rates. We analysed large and small species (above and below 21 kg) separately to assess whether they underwent different extinction dynamics. Following previously demonstrated analytical protocols (Pimiento et al. 2017), we first estimated lineage origination and extinction times and preservation rates and then used the inferred lineage lifespans to fit different extinction models.

We modelled the fossilization and sampling as a Poisson process with preservation rate indicating the expected number of fossil occurrences per sampled lineage per unit of time (1 Myr). Since the available fossil occurrences in our data range between 4.36 and 0.99 Ma but species’ true ranges likely extend beyond those boundaries, we expected the preservation rate to be highest within that time window. Thus, we modelled preservation as a time-variable process with three independent preservation rates, delimited by times of rate shift set at 4.36 and 0.99 Ma. We specified a shared exponential prior distribution on the preservation rates, with rate parameter lambda. To reduce the risk of over-parameterization, we treated lambda as a free hyper-parameter and estimated it from the data, after assigning it a gamma hyper-prior (Gelman et al. 2013).

Times of origination and extinction for each species were estimated jointly with the preservation rates and used in the subsequent extinction rate analyses. Because the analyses are based on regional rather than global occurrence data, the origination of a species might be the result of either a true speciation or a migration event of a species that originated elsewhere. Similarly, we do not distinguish between global or local extinction of a species. All the species that we score as extinct are also globally extinct now, but we cannot with certainty determine their global extinction dates in other parts of Africa due to the incompleteness of the fossil record. We note, however, that hominins were consistently present in our sample throughout the period analysed, whereas they arrived later in other parts of Africa. Survival of carnivore species outside the sample area would therefore not weaken our conclusions.

Posterior estimates of the parameters were sampled using Markov Chain Monte Carlo (MCMC). We ran 20,000,000 MCMC iterations to achieve convergence and adequate sample sizes for all parameters (i.e. effective sample size > 200).

The Pliocene and early Pleistocene mammalian community contained multiple elements without any clear modern analogues, including a diverse array of otters (Mustelidae: Lutrinae), ranging in size from less than 5 kg to over 200 kg (Table S2), associated with freshwater ecosystems (Valenciano et al. 2017). Patterns of change in river systems through time is complex and difficult to capture in a single variable. It may be argued that the drying of rivers, or frequent drastic changes in watershed structure, could lead to the extinction of freshwater-associated faunas, including otters. We tested this possibility by removing otters from our analysis, but our conclusions remain unchanged. Without otters, we still find strong statistical support for a positive correlation between extinction rates of large terrestrial carnivores and average hominin brain size, outperforming climate-driven and time-variable extinction rates models (Table S3). Thus, although hominins may have affected extinction patterns in otters, given evidence for fish consumption nearly 2 Ma (Braun et al. 2010), freshwater-associated organisms could not drive the extinction patterns documented across all carnivores.

Extinction models

We used a sample of posterior estimates of origination and extinction times to fit alternative extinction models. The birth-death models implemented in PyRate include two components: origination and extinction. The former quantifies the rates of taxonomic diversification (through either speciation or immigration, since we consider here regional rather than global fauna) and the latter measures the extinction rate, i.e. the pace at which lineages go (at least locally) extinct. In the following analyses, we assumed constant origination rates and allowed extinction rates to vary through time. We chose to focus our hypothesis testing on extinction rate changes because we aimed to explicitly test possible reasons for the demise of carnivore species in the region. Additionally, simulations have shown that very low preservation rates (e.g. herein preservation prior to 4.36 Ma) may alter the initial estimated speciation rates (Pires et al. 2015). The PyRate analytical framework allows us to carry out model testing for extinction independently of the speciation mode. Indeed, the likelihood of the data is computed as a product between the birth likelihood and the death likelihood (eq. 11 in (Silvestro et al. 2014b)), and speciation and extinction rates can be estimated independently of one another for a given set of origination and extinction times (in this case inferred from the analyses described in the paragraph above). Thus, we hereafter refer to the birth-death models listed below as ‘extinction models’.

We tested three categories of extinction models:

1. models with rate shifts, to provide non-mechanistic null models where rate variation through time is allowed but is not attributed to any known correlated variable;
2. models with environmentally driven rate, to assess whether changes in local palaeoclimate and palaeo-vegetation proxies can adequately explain changes in extinction rates;
3. models with rates driven by changes in hominin mean brain size, to test for a causal link between hominin evolution and changes in carnivore extinction rates.

The first category of models describes instances in which extinction rates change at estimated shift times. Under these models we estimate the number of rate shifts, their temporal placement, and the rates in-between. We tested models with no shift (i.e. constant rates), one shift and two shifts (i.e., two and three rates, respectively). We did not test for higher numbers of shifts because the two-shift model did not obtain a substantially higher marginal likelihood in our model tests (see below) suggesting that additional shifts would not improve the fit of the model. We used uniform priors on the placement of rate shifts and exponential priors on the extinction rates, with rate parameter set to one.

In the second category of models, extinction can respond to climatic variables and vegetation based on linear or exponential correlations (Gjesfjeld et al. 2016). Under these models, the sign and effect size of the correlations along with baseline extinction rates are estimated from the data as free parameters. We tested linear and exponential correlation models for each variable, namely the estimated mean annual temperature, the estimated mean annual precipitation, and the fraction of forest cover. Under the linear correlation model, the extinction rate at any given value *v* of the variable (e.g. temperature) is defined as:

whereas under the exponential model, extinction rate is determined by:

Thus, when the correlation parameters (*g* and α for linear and exponential models, respectively) equal 0, the extinction rate is constant through time and independent of the variables. Posterior estimates of correlation parameters can otherwise indicate positive (when *g* > 0 or α > 0) or negative (when *g* < 0 or α < 0) effects of the variable on extinction rate.

The third category of models describes extinction as a linear or exponential function of estimated hominin mean brain size through time. Since we found support for a correlation between brain size and extinction rates in large carnivores, we explored additional models where changes in brain size had non-zero effects only after a time threshold (see main text). To achieve this, we implemented a new model where a threshold time is estimated as a free parameter. Under this model the extinction rate is constant prior to the threshold, while it varies based on the correlation with brain size after the threshold. We used a uniform prior on the threshold parameter.

To standardise the parameter space and ensure that all comparisons are independent of measurement units, we rescaled all the time-continuous variables (climatic variables and brain size) to range between 0 and 1 within the analysed time window (Lehtonen et al. 2017). We set an exponential prior with rate equal to one on the baseline extinction rate (μ_0_) and uniform priors on the correlation parameters *g* and α.

The fit of each model was quantified by marginal likelihood, which we computed using thermodynamic integration (Lartillot & Philippe 2006) as implemented in PyRate. The use of reasonably informative priors is essential to yielding a good convergence of the thermodynamic integration algorithm. To determine suitable boundaries of the prior on *g* and α, we first ran initial MCMCs on each variable using an arbitrarily large prior, U[-100, 100]. We then defined narrower priors U[-5,5], after verifying that all posterior samples of the correlation parameters fell well within this range. We ran 5,000,000 MCMC iterations using 10 beta-distributed scaling factors (Lehtonen et al. 2017) to compute marginal likelihoods of all models and compared them using Bayes factors (logBF).

We compared the fit between rate shift models (category 1) and correlation models (i.e. linear or exponential) for each time-continuous variable (categories 2 and 3) to assess whether time, climate or hominin brain size best explains extinction patterns in small and large carnivores. We used standard logBF thresholds (Kass & Raftery 1995) to determine the strength of support for the best model against the others. In the case of models receiving very similar support (logBF < 2) we considered the simplest model, i.e. the one with a smaller number of free parameters, as the best one.

All the input data, software, and detailed analysis settings are available as supporting materials. PyRate is an open-source program available here: https://github.com/dsilvestro/PyRate.

Model testing

We found contrasting results between small and large carnivores. In small carnivores, we did not detect strong evidence of changes in extinction rates over the past 4 Myr. Although the highest marginal likelihood was obtained by the birth-death model with two shifts, the constant rate model received an almost identical support (logBF = 0.25; Table S6), indicating that rate variation does not significantly improve the fit. Analyses based solely on terrestrial small carnivores yielded congruent results. Large carnivores underwent an increase in extinction rate through time, and this rate variation is best explained by a strong linear correlation with the log-transformed hominin brain size. This model significantly outperformed all alternatives with logBF > 10 (very strong evidence) against climate-driven extinction models and logBF > 2 (positive evidence) against extinction models with rate shifts. We did not find support for a delayed effect of hominins on extinction rates, with logBF > 2 (positive evidence) against threshold models.

Parameter estimation

Estimated preservation rates were higher for large carnivores than for small ones (Table S7), and for both groups they were, as expected, highest between 4.36 and 0.99 Ma, when the occurrences in our data are found. The higher preservation rate of larger carnivores we observe completely matches our expectations. This is both because the greater robustness of the teeth and bones of the larger species increases the taphonomic preservation rate and because field work is most frequently based on surface collection where larger fossils are more noticeable.

The dynamics and magnitude of extinction rates estimated under the best fitting models differed between small and large carnivores (see Tables S6, S8 for parameter estimates under all models). Extinction rates (quantifying the expected number extinction events per lineage per Myr) in the mid-Pliocene were significantly higher for small carnivores (0.71, 95% CI: 0.52–0.92) than for large ones (0.05, 95% CI: 0.01–0.23). However, while extinction rates in small carnivores remained constant throughout the Pliocene and Quaternary, they strongly increased through time in large carnivores. By the end of the Pliocene, the extinction rate in large carnivores had increased almost 7-fold to 0.36 (95% CI: 0.23–0.51). Around 2 Ma the extinction rates of large and small carnivores were approximately equal and by the Middle Pleistocene the extinction rates of large carnivores further increased to 1.46 (95% CI: 0.87–2.09), thus being twice as high as for small carnivores.

The pattern of rate increase in large carnivores is the result of a strong positive linear correlation with mean hominin brain size, which increased from ca. 400 cm^3^ to >1,500 cm^3^ through time (Table S1; Fig. 1a). The posterior samples of the correlation parameter did not include 0, indicating that the correlation is highly significant.

**Supplementary discussion**

Pattern by sub-guild

The 36 large carnivores identified in the fossil record and included in our study can be broken down into six groups with different diversity trajectories (Fig S1). Conical-toothed cats, dogs and hyenas, which are still present in the area today, and saber-toothed cats, giant otters and giant omnivores, which are regionally or globally extinct. These six groups show different diversity dynamics through time, as discussed below. We note that we here focus on diversity patterns whereas in the main text, we focus on extinction rates. The changes in diversity we report here are therefore not directly related to the changes in extinction rates in figure 1. The initial anthropogenic effects may be associated more with faunal turnover and the later mainly with diversity declines. The diversity declines we discuss here may therefore manifest slightly later than the extinction rate dynamics we show in figure 1, and in particular the time of diversity decline (Fig. S1) will be different from the time of increased extinction rate (Fig. 1).

The group of omnivores is the only non-taxonomically defined group and consists of a bear (cf. *Agriotherium*), a giant marten (aff. *Plesiogulo*) and three giant civets (*Pseudocivetta ingens*, *Sahelictis* cf. *S. korei* and *Viverra leakeyi*). The diversity of the group appears to show a gradual decline in through the period, starting already at 4 Ma. This could mechanistically make sense, since this group of species would have been in direct competition with likely omnivorous hominins throughout the studied time period.

While we did not see any support for the climate hypothesis for the overall pattern we should note that the closest contemporary relatives of at least the giant marten and the bear are associated with forested environments. The phylogenetic placement of the extinct viverrids is poorly known and it is less clear if their closest extant relatives are associated to forested environments like most species in the family or to open environments like the largest extant species in Africa (*Civettictis civetta*). Viewed in isolation, the decline in omnivore diversity could be linked to the reduction in forest cover (Cerling et al. 2011). This association to forested environments could, however, also be a consequence of lower hominin presence in such environments. For example, bears were one of the groups with the highest extinction rate in the Late Pleistocene, where five out of the 13 species alive then died out as part of the megafauna extinction (Sandom et al. 2014). The current association of bears to forested environments may therefore just represent spatial patterns in anthropogenic extinction rate.

The second group to disappear from the region was giant otters. Four species of this group were present during different time periods (*Enhydriodon afman*, *Enhydriodon dikikae*, *Enhydriodon ekecaman* and *Enhydriodon sp. nov.*). The diversity of this group appears to decline earlier than all other groups except the omnivores, and the last record of any of the giant otters is dated at 1.88-2.00 Ma. Although the exact diet of these otters is not known, there are records of the consumption of aquatic resources by hominins near the time of extinction of this group (Braun et al. 2010) and anthropogenically mediated extinctions may be plausible both due to kleptoparasitism and to direct competition over prey items.

The third group that completely disappeared from the region was the saber-toothed cats. This group was highly successful in the area during the time encompassed by this study, with a total of nine species in three genera (*Dinofelis aronoki*, *Dinofelis petteri*, *Dinofelis piveteaui*, *Dinofelis* sp., *Homotherium* sp., *Megantereon* cf. *M. whitei*, *Megantereon ekidoit*, *Megantereon* sp., *Megantereon whitei*). There were four or more species present throughout most of the time period but after a small peak around 2 Ma (which is associated with shifts in species within genera and could be a taxonomic artefact) there was a sharp decline starting around 1.5 Ma and the entire clade was extinct in Africa around 1 Ma. The steep decline of this group appears to have started slightly later than the decline of other groups. This later decline may solely be a function of the analyses focusing on species diversity rather than abundance, however. Analysis of abundance indicates that *Homotherium* (the most readily identified carnivore in the African fossil record) went from being common in Pliocene assemblages at Koobi Fora to a handful of specimens in early Pleistocene assemblages, to only a single bone in the Okote Member at Koobi Fora, which is also the youngest record of the genus in Africa (Werdelin & Lewis 2013b). *Homotherium* survived in the New World until the late Pleistocene and went extinct there around the time of human arrival (Sandom et al. 2014), which could further suggest an anthropogenic causation for the extinction this clade.

The fourth group (the hyenas) are still found in the area but at substantially reduced diversity. Ten extinct species from six genera (*Pachycrocuta brevirostris,* cf. *Parahyaena* sp., *Chasmaporthetes* sp., *Crocuta dietrichi*, *Crocuta eturono*, *Crocuta ultra*, *Hyaena* cf. *H. makapani*, *Hyaena* sp., *Ikelohyaena abronia* and *Parahyaena howelli*) are found in the fossil sites along with a single extant one (*Hyaena hyaena*) and a twelfth (*Crocuta crocuta*), which likely replaced an extinct congeneric species within the last million years. Hyenas had a near constant diversity from around 4 Ma to around 2 Ma when they were the most diverse carnivore group in the system, but they suffered drastic decline from 2 to 1 Ma.

The disappearance of the spotted hyena (*Crocuta crocuta*) from Eurasia in the Late Pleistocene (and therefore the reduction in continental diversity from two to one species) has been investigated through species distribution modelling which found a climatic causation to be extremely unlikely (Varela et al. 2010), and this could potentially further argue against a climatic causation for the reduction in hyena diversity in Africa.

The fifth group includes conical toothed cats and appears to show only slight fluctuations in diversity. There is potentially a slight decline from 2 to 1 Ma but this is could all be anagenic evolution with three separate lineages being present from the beginning of the sampling period until the present (lions evolved from *Panthera* cf *P. leo* to *P. leo*, leopards from *Panthera* cf *P. pardus* to *P. pardus* and cheetahs may have evolved from cf. *Acinonyx* sp to *Acinonyx* sp., and sometime within the last million years to *Acinonyx jubatus*).

The sixth group encompasses the dogs, which also show no signs of decline in diversity. A single species appeared (*Xenocyon falconeri*) around 1.5 Ma (as the only regionally new genus of large carnivores within the last 2 Myr) and was likely replaced in the region by the African wild dog (*Lycaon pictus*) within the last million years.

Since both *Homo* and *Xenocyon* appear within the last 2 Myr, it could in principle be argued that part of the diversity decline we attribute to hominins was driven by canids instead but this seems extremely unlikely given that wild dogs are not a dominant carnivore and actually often suffer substantially from kleptoparasitism and other interactions with the remaining carnivores (Carbone et al. 2006).

When investigating the dynamics of the large carnivores in more detail, it is thus striking that within the group we do not see any clade with any large increase in importance in the system. This is in stark contrast to biotic (non-anthropogenic) driven scenarios of faunal turnover (Silvestro et al. 2015) and it also seems unlikely with regard to climatic causation. Changing climate, at least for shifts between non-extreme climatic conditions, ought to be beneficial for some subguilds and detrimental for others, as opposed to being consistently detrimental. Investigating patterns within subguilds further thus only strengthens our claim of a hominin causation of the elevated extinction rates among large African carnivores.

**Additional references**

Benson-Amram, S., Dantzer, B., Stricker, G., Swanson, E.M. & Holekamp, K.E. (2016) Brain size predicts problem-solving ability in mammalian carnivores. *Proc. Natl. Acad. Sci. U.S.A.* 113, 2532–2537.

Braun, D.R.. Harris, J.W.K., Levin, N.E., McCoy, J.T., Herries, A.I.R., Bamford, M.K.. Bishop, L.C., Richmond, B.G. & Kibunjia, M. (2010) Early hominin diet included diverse terrestrial and aquatic animals 1.95 Ma in East Turkana, Kenya. *Proc. Natl. Acad. Sci. U.S.A.* 107, 10002–10007.

Carbone, C., Frame, L., Frame, G., Malcolm, J., Fanshawe, J., FitzGibbon, C., Schaller, G., Gordon, I.J., Rowcliffe, J.M. & du Toit, J.T. (2006) Feeding success of African wild dogs (*Lycaon pictus*) in the Serengeti: the Effects of group size and kleptoparasitism. *J. Zool.* 266, 153–161.

Dirks P.H.G.M. et al. (2017) The age of *Homo naledi* and associated sediments in the Rising Star Cave, South Africa. *eLife* 6, e24231.

Faurby, S.. Eiserhardt, W.L. & Svenning, J.C. (2016) Strong effects of variation in taxonomic opinion on diversification analyses. *Method Ecol. Evol.* 7, 4-13.

Gelman, A., Carlin, J. B., Stern, H.S., Dunson, D.B., Vehtari, A. & Rubin, D.B. (2013) *Bayesian Data Analysis, 3rd edition.* CRC Press, Boca Raton.

Gjesfjeld, E., Chang, J., Silvestro, D., Kelty, C. & Alfaro, M. (2016) Competition and extinction explain the evolution of diversity in American automobiles. *Palgrave Communications* 2, 16038.

Howell, F.C. & Petter, R.G. (1976) “Carnivora from Omo group formations, Southern Ethiopia” In: *Earliest Man and Environments in the Lake Rudolf Basin* (Coppens, Y., Leakey, R.E.F. & Isaac G.L. Eds.) University of Chicago Press, Chicago, pp. 314–331.

Kass, R.E. & Raftery, A.E. (1995) Bayes Factors. *J. Am. Stat. Assoc.* 90, 773–795.

Lartillot, N. & Philippe, H. (2006) Computing Bayes factors using thermodynamic integration. *Syst. Biol.* 55, 195–207.

Lehtonen, S., Silvestro, D., Karger, D.N., Scotese, C., Tuomisto, H., Kessler, M., Peña, C., Wahlberg, N. & Antonelli, A. (2017) Environmentally driven extinction and opportunistic origination explain fern diversification patterns. *Sci. Rep*. 7, 4831.

Petter, G. (1973) “Carnivores pléistocènes du ravin d'Olduvai (Tanzanie)” In: *Fossil Vertebrates of Africa Volume 3* (Leakey, L. S. B. , Savage, R. J. G. & Coryndon S. C. Eds) Academic Press, Cambridge, pp. 44–100.

Pimiento, C., Griffin, J.N., Clements, C.F., Silvestro, D., Varela, S., Uhen, M.D. & Jaramillo, C. (2017) The Pliocene marine megafauna extinction and its impact on functional diversity. *Nat. Ecol. Evol*. 1, 1100–1106.

Pires, M.M., Silvestro, D. & Quental, T.B. (2015) Continental faunal exchange and the asymmetrical radiation of carnivores. *Proc. Royal Soc. B* 282, 20151952.

Silvestro, D., Antonelli, A., Salamin, N. & Quental, T.B. (2015) The role of clade competition in the diversification of North American canids. *Proc. Natl. Acad. Sci. U.S.A.* 112, 8684–8689.

Silvestro, D., Schnitzler, J. , Liow, L.H., Antonelli, A. & Salamin, N. (2014b) Bayesian estimation of speciation and extinction from incomplete fossil occurrence data. *Syst. Biol.* 63, 349–367.

Valenciano, A., Abella, J., Werdelin, L., Atwell, M., Sierra, Á.Á., Morales, J. & Hartstone-Rose, A. (2017) Preliminary approximation on the locomotion and body mass of giant mustelids and other musteloids (Ailuridae and Procyonidae) throughout the Neogene and Quaternary. *Zitteliana* 91, 91.

Werdelin, L. (2003) Carnivores from the Kanapoi hominid site, Turkana Basin, northern Kenya. *Contrib. sci.* 498, 115–132.

Werdelin, L. & Dehghani, R. (2011) “Carnivora” In: *Paleontology and Geology of Laetoli, Tanzania: Human Evolution in Context, Volume 2: Vertebrate Paleobiology and Paleoanthropology Series* (Harrison, R. Ed.). Springer, New York, pp. 189–232.

Werdelin, L. & Lewis, M. E. (2000) Carnivora from the South Turkwel hominid site, northern Kenya. *J. Paleontol.* 74, 1173–1180.

Werdelin, L. & Lewis M.E. (2013b) *Koobi Fora Research Project Volume 7: The Carnivora*. California Academy of Sciences, San Francisco.

Werdelin, L., Lewis, M.E., & Haile-Selassie, Y. (2013) Mid-Pliocene Carnivora from the Woranso-Mille Area, Afar Region, Ethiopia. *J. Mamm. Evol.* 21, 331–347.

Werdelin, L. & Manthi, F.K. (2012) Carnivora from the Kanapoi hominin site, northern Kenya. *J. Afr. Earth Sci.* 64, 1–8.

Wood, B. & Strait, D. (2004) Patterns of resource use in early *Homo* and *Paranthropus*. *J. Hum. Evol.* 46, 119–162.

| **Table S1. Brain size in different hominin specimens from Africa.** The data were compiled from fossil occurrences described by (Seymour et al. 2016) (genera *Homo* and *Australopithecus*) and by (White et al. 2009) (*Ardipithecus ramidus*). | | | |
| --- | --- | --- | --- |
| Species | Brain (cm^3^) | Age (Myr) | |
|  |  | min | max |
| *Homo sapiens* | 1493 | 0 | 0.2 |
| *H. sapiens* | 1493 | 0.018 | 0.023 |
| *H. sapiens* | 1493 | 0.018 | 0.023 |
| *H. sapiens* | 1510 | 0.08 | 0.08 |
| *H. sapiens* | 1367 | 0.12 | 0.12 |
| *H. rudolfensis* | 752 | 1.88 | 1.88 |
| *H. heidelbergensis* | 1100 | 0.4 | 0.4 |
| *H. heidelbergensis* | 1325 | 0.18 | 0.18 |
| *H. erectus* | 691 | 1.55 | 1.55 |
| *H. erectus* | 1067 | 1.4 | 1.4 |
| *H. habilis* | 590 | 1.8 | 1.8 |
| *Australopithecus africanus* | 485 | 2.5 | 2.5 |
| *A. africanus* | 436 | 2.5 | 2.5 |
| *A. africanus* | 435 | 3.1 | 3.1 |
| *A. africanus* | 462 | 2.4 | 2.8 |
| *A. africanus* | 462 | 2.4 | 2.8 |
| *A. africanus* | 462 | 2.4 | 2.8 |
| *A. africanus* | 462 | 2.4 | 2.8 |
| *A. africanus* | 462 | 2.4 | 2.8 |
| *A. afarensis* | 400 | 3.18 | 3.18 |
| *A. afarensis* | 514 | 3.18 | 3.18 |
| *Ardipithecus ramidus* | 325 | 4.4 | 4.4 |

| **Table S2. Analysed fossil species and occurrences** | | | | | | | | | | |
| --- | --- | --- | --- | --- | --- | --- | --- | --- | --- | --- |
| **Table S2a Species** | | | | | | | | | | |
| Family | | Species | Status | | | Aquatic | | | Size | |
| Canidae | | *Aff Otocyon* | Extinct | | | No | | | Small | |
|  | | *Canis sp. A* | Extinct | | | No | | | Small | |
|  | | *Canis sp. B* | Extinct | | | No | | | Small | |
|  | | *Eucyon kuta* | Extinct | | | No | | | Small | |
|  | | *Lupulella sp.* | Extinct | | | No | | | Small | |
|  | | *Nyctereutes barryi* | Extinct | | | No | | | Small | |
|  | | *Nyctereutes lockwoodi* | Extinct | | | No | | | Small | |
|  | | *Prototocyon recki* | Extinct | | | No | | | Small | |
|  | | *Vulpes sp.* | Extinct | | | No | | | Small | |
|  | | *Xenocyon falconeri* | Extinct | | | No | | | Large | |
| Felidae | | *Acinonyx sp. nov.* | Extinct | | | No | | | Large | |
|  | | *Caracal/ Leptailurus sp* | Extinct | | | No | | | Small | |
|  | | *Cf. Acinonyx sp.* | Extinct | | | No | | | Large | |
|  | | *Dinofelis aronoki* | Extinct | | | No | | | Large | |
|  | | *Dinofelis petteri* | Extinct | | | No | | | Large | |
|  | | *Dinofelis piveteaui* | Extinct | | | No | | | Large | |
|  | | *Dinofelis sp.* | Extinct | | | No | | | Large | |
|  | | *Felis sp.* | Extant | | | No | | | Small | |
|  | | *Homotherium sp.* | Extinct | | | No | | | Large | |
|  | | *Megantereon cf. M. whitei* | Extinct | | | No | | | Large | |
|  | | *Megantereon ekidoit* | Extinct | | | No | | | Large | |
|  | | *Megantereon sp.* | Extinct | | | No | | | Large | |
|  | | *Megantereon whitei* | Extinct | | | No | | | Large | |
|  | | *Panthera cf P. leo* | Extinct | | | No | | | Large | |
|  | | *Panthera cf. P. pardus* | Extinct | | | No | | | Large | |
|  | | *Panthera leo* | Extant | | | No | | | Large | |
|  | | *Panthera pardus* | Extant | | | No | | | Large | |
| Herpestidae | | *Atilax sp.* | Extinct | | | No | | | Small | |
|  | | *Cf Ichneumia sp.* | Extinct | | | No | | | Small | |
|  | | *Galerella debilis* | Extinct | | | No | | | Small | |
|  | | *Galerella primitivus* | Extinct | | | No | | | Small | |
|  | | *Galerella sp.* | Extinct | | | No | | | Small | |
|  | | *Helogale hirtula* | Extant | | | No | | | Small | |
|  | | *Helogale kitafe* | Extinct | | | No | | | Small | |
|  | | *Helogale palaeogracilis* | Extinct | | | No | | | Small | |
|  | | *Herpestes ichneumon* | Extant | | | No | | | Small | |
|  | | *Herpestes palaeoserengetensis* | Extinct | | | No | | | Small | |
|  | | *Ichneumia albicauda* | Extant | | | No | | | Small | |
|  | | *Mungos dietrichi* | Extinct | | | No | | | Small | |
|  | | *Mungos sp. nov.* | Extinct | | | No | | | Small | |
| Hyanidae | | *Aff. Proteles sp.* | Extinct | | | No | | | Small | |
|  | | *Cf. Pachycrocuta sp,* | Extinct | | | No | | | Large | |
|  | | *Cf. Parahyaena sp.* | Extinct | | | No | | | Large | |
|  | | *Chasmaporthetes sp.* | Extinct | | | No | | | Large | |
|  | | *Crocuta dietrichi* | Extinct | | | No | | | Large | |
|  | | *Crocuta eturono* | Extinct | | | No | | | Large | |
|  | | *Crocuta ultra* | Extinct | | | No | | | Large | |
|  | | *Hyaena cf. H. makapani* | Extinct | | | No | | | Large | |
|  | | *Hyaena hyaena* | Extant | | | No | | | Large | |
|  | | *Hyaena sp.* | Extinct | | | No | | | Large | |
|  | | *Ikelohyaena abronia* | Extinct | | | No | | | Large | |
|  | | *Lycyaenops cf. L. silberbergi* | Extinct | | | No | | | Small | |
|  | | *Parahyaena howelli* | Extinct | | | No | | | Large | |
| Mustelidae | | *Aff. Ictonyx* | Extinct | | | No | | | Small | |
|  | | *Aff. Plesiogulo* | Extinct | | | No | | | Large | |
|  | | *Aonyx sp.* | Extinct | | | Yes | | | Small | |
|  | | *Aoxini gen. et sp. nov.* | Extinct | | | Yes | | | Small | |
|  | | *Enhydriodon afman* | Extinct | | | Yes | | | Large | |
|  | | *Enhydriodon dikikae* | Extinct | | | Yes | | | Large | |
|  | | *Enhydriodon ekecaman* | Extinct | | | Yes | | | Large | |
|  | | *Enhydriodon sp. E* | Extinct | | | Yes | | | Large | |
|  | | *Hydrictis gudho* | Extinct | | | Yes | | | Small | |
|  | | *Hydrictis sp.* | Extinct | | | Yes | | | Small | |
|  | | *Lutra hearsti* | Extinct | | | Yes | | | Small | |
|  | | *Lutrinae gen et sp. Nov.* | Extinct | | | Yes | | | Small | |
|  | | *Mellivora sp.* | Extinct | | | No | | | Small | |
|  | | *Mustelidae indet A* | Extinct | | | No | | | Small | |
|  | | *Mustelidae indet B* | Extinct | | | No | | | Small | |
|  | | *Mustelidae indet C* | Extinct | | | No | | | Small | |
|  | | *Mustelidae indet C* | Extinct | | | No | | | Small | |
|  | | *Mustelidae indet D* | Extinct | | | No | | | Small | |
|  | | *Prepoecilogale bolti* | Extinct | | | No | | | Small | |
|  | | *Torolutra cf. T. ougandensis* | Extinct | | | Yes | | | Small | |
|  | | *Torolutra sp.* | Extinct | | | Yes | | | Small | |
| Ursidae | | *Cf. Agriotherium sp.* | Extinct | | | No | | | Large | |
| Viverridae | | *Civettictis aff. C. civetta* | Extinct | | | No | | | Small | |
|  | | *Genetta cf. G. servalina* | Extant | | | No | | | Small | |
|  | | *Genetta genetta/ maculata* | Extant | | | No | | | Small | |
|  | | *Genetta nyakitongwer* | Extinct | | | No | | | Small | |
|  | | *Peudocivetta ingens* | Extinct | | | No | | | Large | |
|  | | *Sahelictis cf. S.korei* | Extinct | | | No | | | Large | |
|  | | *Viverra leakeyi* | Extinct | | | No | | | Large | |
|  | | *Viverridae sp. A large* | Extinct | | | No | | | Small | |
|  | | *Viverridae sp. B small* | Extinct | | | No | | | Small | |
|  | | *Viverridae sp. C small* | Extinct | | | No | | | Small | |
|  | | *Viverridae sp. D* | Extinct | | | No | | | Small | |
|  | | *Viverridae sp. E* | Extinct | | | No | | | Small | |
|  | | *Viverridae sp. F* | Extinct | | | No | | | Small | |
|  | | *Viverridae sp. G* | Extinct | | | No | | | Small | |
| **Table S2b records** | | | | | | | | | | |
| Family | Species | | | Status | Max age | | Min age | Aquatic | | Size |
| Canidae | *Aff Otocyon* | | | Extinct | 3.85 | | 3.63 | No | | Small |
|  | *Canis sp. A* | | | Extinct | 3.18 | | 2.92 | No | | Small |
|  | *Canis sp. A* | | | Extinct | 3.22 | | 3.18 | No | | Small |
|  | *Canis sp. A* | | | Extinct | 3.4 | | 3.28 | No | | Small |
|  | *Canis sp. A* | | | Extinct | 3.5 | | 3.2 | No | | Small |
|  | *Canis sp. A* | | | Extinct | 3.85 | | 3.63 | No | | Small |
|  | *Canis sp. B* | | | Extinct | 3.85 | | 3.63 | No | | Small |
|  | *Eucyon kuta* | | | Extinct | 3.7 | | 3.5 | No | | Small |
|  | *Lupulella sp.* | | | Extinct | 1.87 | | 1.7 | No | | Small |
|  | *Lupulella sp.* | | | Extinct | 1.88 | | 1.64 | No | | Small |
|  | *Lupulella sp.* | | | Extinct | 2 | | 1.88 | No | | Small |
|  | *Nyctereutes barryi* | | | Extinct | 3.85 | | 3.63 | No | | Small |
|  | *Nyctereutes lockwoodi* | | | Extinct | 3.18 | | 2.92 | No | | Small |
|  | *Nyctereutes lockwoodi* | | | Extinct | 3.4 | | 3.28 | No | | Small |
|  | *Prototocyon recki* | | | Extinct | 1.87 | | 1.7 | No | | Small |
|  | *Vulpes sp.* | | | Extinct | 1.64 | | 1.39 | No | | Small |
|  | *Vulpes sp.* | | | Extinct | 2 | | 1.88 | No | | Small |
|  | *Xenocyon falconeri* | | | Extinct | 0.99 | | 0.99 | No | | Large |
|  | *Xenocyon falconeri* | | | Extinct | 1.7 | | 1.2 | No | | Large |
| Felidae | *Acinonyx sp. nov.* | | | Extinct | 1.64 | | 1.39 | No | | Large |
|  | *Acinonyx sp. nov.* | | | Extinct | 1.88 | | 1.64 | No | | Large |
|  | *Caracal/ Leptailurus sp.* | | | Extinct | 2.66 | | 2.66 | No | | Small |
|  | *Caracal/ Leptailurus sp.* | | | Extinct | 3.36 | | 2.68 | No | | Small |
|  | *Caracal/ Leptailurus sp.* | | | Extinct | 3.4 | | 3.28 | No | | Small |
|  | *Caracal/ Leptailurus sp.* | | | Extinct | 3.85 | | 3.63 | No | | Small |
|  | *Cf. Acinonyx sp.* | | | Extinct | 2 | | 1.88 | No | | Large |
|  | *Cf. Acinonyx sp.* | | | Extinct | 2.85 | | 2.52 | No | | Large |
|  | *Cf. Acinonyx sp.* | | | Extinct | 3.07 | | 3 | No | | Large |
|  | *Cf. Acinonyx sp.* | | | Extinct | 3.85 | | 3.63 | No | | Large |
|  | *Dinofelis aronoki* | | | Extinct | 1.88 | | 1.64 | No | | Large |
|  | *Dinofelis aronoki* | | | Extinct | 1.9 | | 1.6 | No | | Large |
|  | *Dinofelis aronoki* | | | Extinct | 2 | | 1.88 | No | | Large |
|  | *Dinofelis aronoki* | | | Extinct | 3.18 | | 2.92 | No | | Large |
|  | *Dinofelis aronoki* | | | Extinct | 3.36 | | 2.68 | No | | Large |
|  | *Dinofelis aronoki* | | | Extinct | 4.1 | | 3.36 | No | | Large |
|  | *Dinofelis petteri* | | | Extinct | 2.52 | | 2.37 | No | | Large |
|  | *Dinofelis petteri* | | | Extinct | 2.66 | | 2.66 | No | | Large |
|  | *Dinofelis petteri* | | | Extinct | 2.85 | | 2.52 | No | | Large |
|  | *Dinofelis petteri* | | | Extinct | 3.22 | | 3.18 | No | | Large |
|  | *Dinofelis petteri* | | | Extinct | 3.36 | | 2.68 | No | | Large |
|  | *Dinofelis petteri* | | | Extinct | 3.36 | | 2.85 | No | | Large |
|  | *Dinofelis petteri* | | | Extinct | 3.36 | | 3.21 | No | | Large |
|  | *Dinofelis petteri* | | | Extinct | 3.4 | | 3.28 | No | | Large |
|  | *Dinofelis petteri* | | | Extinct | 3.85 | | 3.63 | No | | Large |
|  | *Dinofelis petteri* | | | Extinct | 3.9 | | 3.7 | No | | Large |
|  | *Dinofelis petteri* | | | Extinct | 4.23 | | 4.07 | No | | Large |
|  | *Dinofelis piveteaui* | | | Extinct | 1 | | 0.9 | No | | Large |
|  | *Dinofelis piveteaui* | | | Extinct | 1.64 | | 1.39 | No | | Large |
|  | *Dinofelis sp.* | | | Extinct | 1.7 | | 1.2 | No | | Large |
|  | *Dinofelis sp.* | | | Extinct | 1.87 | | 1.7 | No | | Large |
|  | *Felis sp.* | | | Extant | 2 | | 1.8 | No | | Small |
|  | *Felis sp.* | | | Extant | 3.07 | | 3 | No | | Small |
|  | *Felis sp.* | | | Extant | 3.22 | | 3.18 | No | | Small |
|  | *Felis sp.* | | | Extant | 3.36 | | 2.85 | No | | Small |
|  | *Felis sp.* | | | Extant | 3.4 | | 3.28 | No | | Small |
|  | *Felis sp.* | | | Extant | 3.85 | | 3.63 | No | | Small |
|  | *Homotherium sp.* | | | Extinct | 1.64 | | 1.39 | No | | Large |
|  | *Homotherium sp.* | | | Extinct | 1.88 | | 1.6 | No | | Large |
|  | *Homotherium sp.* | | | Extinct | 1.88 | | 1.64 | No | | Large |
|  | *Homotherium sp.* | | | Extinct | 1.9 | | 1.6 | No | | Large |
|  | *Homotherium sp.* | | | Extinct | 2 | | 1.8 | No | | Large |
|  | *Homotherium sp.* | | | Extinct | 2 | | 1.88 | No | | Large |
|  | *Homotherium sp.* | | | Extinct | 2.33 | | 1.88 | No | | Large |
|  | *Homotherium sp.* | | | Extinct | 2.37 | | 2.33 | No | | Large |
|  | *Homotherium sp.* | | | Extinct | 2.66 | | 2.66 | No | | Large |
|  | *Homotherium sp.* | | | Extinct | 2.85 | | 2.52 | No | | Large |
|  | *Homotherium sp.* | | | Extinct | 3 | | 2.53 | No | | Large |
|  | *Homotherium sp.* | | | Extinct | 3 | | 3 | No | | Large |
|  | *Homotherium sp.* | | | Extinct | 3.07 | | 3 | No | | Large |
|  | *Homotherium sp.* | | | Extinct | 3.18 | | 2.92 | No | | Large |
|  | *Homotherium sp.* | | | Extinct | 3.21 | | 3 | No | | Large |
|  | *Homotherium sp.* | | | Extinct | 3.22 | | 3.18 | No | | Large |
|  | *Homotherium sp.* | | | Extinct | 3.36 | | 2.68 | No | | Large |
|  | *Homotherium sp.* | | | Extinct | 3.36 | | 3.21 | No | | Large |
|  | *Homotherium sp.* | | | Extinct | 3.4 | | 3.28 | No | | Large |
|  | *Homotherium sp.* | | | Extinct | 3.5 | | 3.36 | No | | Large |
|  | *Homotherium sp.* | | | Extinct | 3.7 | | 3.5 | No | | Large |
|  | *Homotherium sp.* | | | Extinct | 3.85 | | 3.63 | No | | Large |
|  | *Homotherium sp.* | | | Extinct | 3.9 | | 3.7 | No | | Large |
|  | *Homotherium sp.* | | | Extinct | 4.1 | | 3.36 | No | | Large |
|  | *Homotherium sp.* | | | Extinct | 4.23 | | 4.07 | No | | Large |
|  | *Homotherium sp.* | | | Extinct | 4.35 | | 4.1 | No | | Large |
|  | *Megantereon cf. M. whitei* | | | Extinct | 3.36 | | 2.68 | No | | Large |
|  | *Megantereon ekidoit* | | | Extinct | 3.5 | | 3.2 | No | | Large |
|  | *Megantereon sp.* | | | Extinct | 2.33 | | 1.88 | No | | Large |
|  | *Megantereon sp.* | | | Extinct | 2.37 | | 2.33 | No | | Large |
|  | *Megantereon sp.* | | | Extinct | 2.52 | | 2.37 | No | | Large |
|  | *Megantereon sp.* | | | Extinct | 2.85 | | 2.52 | No | | Large |
|  | *Megantereon sp.* | | | Extinct | 3.07 | | 3 | No | | Large |
|  | *Megantereon sp.* | | | Extinct | 3.36 | | 2.85 | No | | Large |
|  | *Megantereon whitei* | | | Extinct | 1.64 | | 1.39 | No | | Large |
|  | *Megantereon whitei* | | | Extinct | 1.88 | | 1.64 | No | | Large |
|  | *Megantereon whitei* | | | Extinct | 2 | | 1.88 | No | | Large |
|  | *Panthera cf P. leo* | | | Extinct | 3.7 | | 3.5 | No | | Large |
|  | *Panthera cf P. leo* | | | Extinct | 3.85 | | 3.63 | No | | Large |
|  | *Panthera cf. P. pardus* | | | Extinct | 2.66 | | 2.66 | No | | Large |
|  | *Panthera cf. P. pardus* | | | Extinct | 3.85 | | 3.63 | No | | Large |
|  | *Panthera leo* | | | Extant | 1.39 | | 1.12 | No | | Large |
|  | *Panthera leo* | | | Extant | 1.64 | | 1.39 | No | | Large |
|  | *Panthera leo* | | | Extant | 1.7 | | 1.2 | No | | Large |
|  | *Panthera leo* | | | Extant | 1.87 | | 1.7 | No | | Large |
|  | *Panthera leo* | | | Extant | 1.88 | | 1.64 | No | | Large |
|  | *Panthera leo* | | | Extant | 2 | | 1.88 | No | | Large |
|  | *Panthera pardus* | | | Extant | 1.7 | | 1.2 | No | | Large |
|  | *Panthera pardus* | | | Extant | 1.87 | | 1.7 | No | | Large |
|  | *Panthera pardus* | | | Extant | 1.88 | | 1.64 | No | | Large |
|  | *Panthera pardus* | | | Extant | 2 | | 1.88 | No | | Large |
|  | *Panthera pardus* | | | Extant | 2.33 | | 1.88 | No | | Large |
|  | *Panthera pardus* | | | Extant | 2.85 | | 2.52 | No | | Large |
|  | *Panthera pardus* | | | Extant | 3.07 | | 3 | No | | Large |
|  | *Panthera pardus* | | | Extant | 3.36 | | 2.85 | No | | Large |
| Herpestidae | *Atilax sp.* | | | Extinct | 1.7 | | 1.2 | No | | Small |
|  | *Cf Ichneumia sp.* | | | Extinct | 3.7 | | 3.5 | No | | Small |
|  | *Galerella debilis* | | | Extinct | 1.87 | | 1.7 | No | | Small |
|  | *Galerella primitivus* | | | Extinct | 1.87 | | 1.7 | No | | Small |
|  | *Galerella sp.* | | | Extinct | 3.85 | | 3.63 | No | | Small |
|  | *Helogale hirtula* | | | Extant | 2.33 | | 1.88 | No | | Small |
|  | *Helogale hirtula* | | | Extant | 2.37 | | 2.33 | No | | Small |
|  | *Helogale kitafe* | | | Extinct | 2.85 | | 2.52 | No | | Small |
|  | *Helogale kitafe* | | | Extinct | 3.36 | | 2.85 | No | | Small |
|  | *Helogale palaeogracilis* | | | Extinct | 2.66 | | 2.66 | No | | Small |
|  | *Helogale palaeogracilis* | | | Extinct | 3.85 | | 3.63 | No | | Small |
|  | *Helogale palaeogracilis* | | | Extinct | 4.36 | | 3.85 | No | | Small |
|  | *Herpestes ichneumon* | | | Extant | 1.87 | | 1.7 | No | | Small |
|  | *Herpestes ichneumon* | | | Extant | 3.85 | | 3.63 | No | | Small |
|  | *Herpestes palaeoserengetensis* | | | Extinct | 3.85 | | 3.63 | No | | Small |
|  | *Ichneumia albicauda* | | | Extant | 1.87 | | 1.7 | No | | Small |
|  | *Mungos dietrichi* | | | Extinct | 1.87 | | 1.7 | No | | Small |
|  | *Mungos dietrichi* | | | Extinct | 2.66 | | 2.66 | No | | Small |
|  | *Mungos dietrichi* | | | Extinct | 3 | | 2.53 | No | | Small |
|  | *Mungos dietrichi* | | | Extinct | 3.21 | | 3 | No | | Small |
|  | *Mungos dietrichi* | | | Extinct | 3.85 | | 3.63 | No | | Small |
|  | *Mungos minutus* | | | Extinct | 1.87 | | 1.7 | No | | Small |
|  | *Mungos sp. Nov.* | | | Extinct | 3.85 | | 3.63 | No | | Small |
| Hyanidae | *Aff. Proteles sp.* | | | Extinct | 4.36 | | 3.85 | No | | Small |
|  | *Cf. Pachycrocuta sp,* | | | Extinct | 3.21 | | 3 | No | | Large |
|  | *Cf. Pachycrocuta sp,* | | | Extinct | 3.36 | | 3.21 | No | | Large |
|  | *Cf. Pachycrocuta sp,* | | | Extinct | 3.5 | | 3.2 | No | | Large |
|  | *Cf. Pachycrocuta sp,* | | | Extinct | 3.85 | | 3.63 | No | | Large |
|  | *Cf. Pachycrocuta sp,* | | | Extinct | 4.1 | | 3.36 | No | | Large |
|  | *Cf. Parahyaena sp.* | | | Extinct | 1.88 | | 1.64 | No | | Large |
|  | *Cf. Parahyaena sp.* | | | Extinct | 1.88 | | 1.64 | No | | Large |
|  | *Chasmaporthetes sp.* | | | Extinct | 3.22 | | 3.18 | No | | Large |
|  | *Chasmaporthetes sp.* | | | Extinct | 3.4 | | 3.28 | No | | Large |
|  | *Chasmaporthetes sp.* | | | Extinct | 3.7 | | 3.5 | No | | Large |
|  | *Chasmaporthetes sp.* | | | Extinct | 3.9 | | 3.7 | No | | Large |
|  | *Crocuta dietrichi* | | | Extinct | 2 | | 1.88 | No | | Large |
|  | *Crocuta dietrichi* | | | Extinct | 2.33 | | 1.88 | No | | Large |
|  | *Crocuta dietrichi* | | | Extinct | 2.37 | | 2.33 | No | | Large |
|  | *Crocuta dietrichi* | | | Extinct | 2.66 | | 2.66 | No | | Large |
|  | *Crocuta dietrichi* | | | Extinct | 3.07 | | 3 | No | | Large |
|  | *Crocuta dietrichi* | | | Extinct | 3.22 | | 3.18 | No | | Large |
|  | *Crocuta dietrichi* | | | Extinct | 3.36 | | 3.21 | No | | Large |
|  | *Crocuta dietrichi* | | | Extinct | 3.4 | | 3.28 | No | | Large |
|  | *Crocuta dietrichi* | | | Extinct | 3.5 | | 3.2 | No | | Large |
|  | *Crocuta dietrichi* | | | Extinct | 3.5 | | 3.36 | No | | Large |
|  | *Crocuta dietrichi* | | | Extinct | 3.7 | | 3.5 | No | | Large |
|  | *Crocuta dietrichi* | | | Extinct | 3.85 | | 3.63 | No | | Large |
|  | *Crocuta dietrichi* | | | Extinct | 4 | | 3.4 | No | | Large |
|  | *Crocuta eturono* | | | Extinct | 1.88 | | 1.64 | No | | Large |
|  | *Crocuta eturono* | | | Extinct | 2 | | 1.88 | No | | Large |
|  | *Crocuta eturono* | | | Extinct | 3.18 | | 2.92 | No | | Large |
|  | *Crocuta eturono* | | | Extinct | 3.22 | | 3.18 | No | | Large |
|  | *Crocuta eturono* | | | Extinct | 3.36 | | 2.68 | No | | Large |
|  | *Crocuta eturono* | | | Extinct | 4.1 | | 3.36 | No | | Large |
|  | *Crocuta ultra* | | | Extinct | 0.99 | | 0.99 | No | | Large |
|  | *Crocuta ultra* | | | Extinct | 1.64 | | 1.39 | No | | Large |
|  | *Crocuta ultra* | | | Extinct | 1.7 | | 1.2 | No | | Large |
|  | *Crocuta ultra* | | | Extinct | 1.87 | | 1.7 | No | | Large |
|  | *Crocuta ultra* | | | Extinct | 1.88 | | 1.64 | No | | Large |
|  | *Crocuta ultra* | | | Extinct | 2 | | 1.88 | No | | Large |
|  | *Crocuta ultra* | | | Extinct | 2.33 | | 1.88 | No | | Large |
|  | *Crocuta ultra* | | | Extinct | 2.35 | | 1.88 | No | | Large |
|  | *Crocuta ultra* | | | Extinct | 2.52 | | 2.35 | No | | Large |
|  | *Hyaena cf. H. makapani* | | | Extinct | 1.88 | | 1.64 | No | | Large |
|  | *Hyaena cf. H. makapani* | | | Extinct | 2 | | 1.88 | No | | Large |
|  | *Hyaena cf. H. makapani* | | | Extinct | 3.07 | | 3 | No | | Large |
|  | *Hyaena cf. H. makapani* | | | Extinct | 3.36 | | 2.85 | No | | Large |
|  | *Hyaena hyaena* | | | Extant | 1.7 | | 1.2 | No | | Large |
|  | *Hyaena hyaena* | | | Extant | 1.87 | | 1.7 | No | | Large |
|  | *Hyaena hyaena* | | | Extant | 1.88 | | 1.6 | No | | Large |
|  | *Hyaena hyaena* | | | Extant | 1.88 | | 1.64 | No | | Large |
|  | *Hyaena hyaena* | | | Extant | 2.33 | | 1.88 | No | | Large |
|  | *Hyaena sp.* | | | Extinct | 2.35 | | 1.88 | No | | Large |
|  | *Hyaena sp.* | | | Extinct | 2.37 | | 2.33 | No | | Large |
|  | *Hyaena sp.* | | | Extinct | 2.52 | | 2.37 | No | | Large |
|  | *Hyaena sp.* | | | Extinct | 3.36 | | 2.68 | No | | Large |
|  | *Hyaena sp.* | | | Extinct | 3.5 | | 3.36 | No | | Large |
|  | *Hyaena sp.* | | | Extinct | 4.35 | | 4.1 | No | | Large |
|  | *Ikelohyaena abronia* | | | Extinct | 2.66 | | 2.66 | No | | Large |
|  | *Ikelohyaena abronia* | | | Extinct | 3.4 | | 3.28 | No | | Large |
|  | *Ikelohyaena abronia* | | | Extinct | 3.85 | | 3.63 | No | | Large |
|  | *Lycyaenops cf. L. silberbergi* | | | Extinct | 3.85 | | 3.63 | No | | Small |
|  | *Parahyaena howelli* | | | Extinct | 3.85 | | 3.63 | No | | Large |
|  | *Parahyaena howelli* | | | Extinct | 3.9 | | 3.7 | No | | Large |
|  | *Parahyaena howelli* | | | Extinct | 4.23 | | 4.07 | No | | Large |
|  | *Parahyaena howelli* | | | Extinct | 4.36 | | 3.85 | No | | Large |
| Mustelidae | *Aff. Ictonyx* | | | Extinct | 3.36 | | 2.68 | No | | Small |
|  | *Aff. Plesiogulo* | | | Extinct | 3.36 | | 2.68 | No | | Large |
|  | *Aonyx sp.* | | | Extinct | 1.7 | | 1.2 | Yes | | Small |
|  | *Aonyx sp.* | | | Extinct | 1.87 | | 1.7 | Yes | | Small |
|  | *Aonyx sp.* | | | Extinct | 1.88 | | 1.64 | Yes | | Small |
|  | *Aoxini gen. et sp. nov.* | | | Extinct | 4.36 | | 3.85 | Yes | | Small |
|  | *Enhydriodon afman* | | | Extinct | 2 | | 1.88 | Yes | | Large |
|  | *Enhydriodon afman* | | | Extinct | 3 | | 3 | Yes | | Large |
|  | *Enhydriodon afman* | | | Extinct | 3.22 | | 3.18 | Yes | | Large |
|  | *Enhydriodon afman* | | | Extinct | 3.36 | | 2.68 | Yes | | Large |
|  | *Enhydriodon afman* | | | Extinct | 3.36 | | 3.21 | Yes | | Large |
|  | *Enhydriodon afman* | | | Extinct | 3.4 | | 3.28 | Yes | | Large |
|  | *Enhydriodon afman* | | | Extinct | 3.5 | | 3.36 | Yes | | Large |
|  | *Enhydriodon afman* | | | Extinct | 4.35 | | 4.1 | Yes | | Large |
|  | *Enhydriodon dikikae* | | | Extinct | 3.22 | | 3.18 | Yes | | Large |
|  | *Enhydriodon dikikae* | | | Extinct | 3.4 | | 3.28 | Yes | | Large |
|  | *Enhydriodon dikikae* | | | Extinct | 4 | | 3.4 | Yes | | Large |
|  | *Enhydriodon dikikae* | | | Extinct | 4.23 | | 4.07 | Yes | | Large |
|  | *Enhydriodon ekecaman* | | | Extinct | 4.23 | | 4.07 | Yes | | Large |
|  | *Enhydriodon sp. E* | | | Extinct | 2.37 | | 2.33 | Yes | | Large |
|  | *Enhydriodon sp. E* | | | Extinct | 2.85 | | 2.52 | Yes | | Large |
|  | *Enhydriodon sp. E* | | | Extinct | 3.07 | | 3 | Yes | | Large |
|  | *Enhydriodon sp. E* | | | Extinct | 3.36 | | 2.85 | Yes | | Large |
|  | *Hydrictis gudho* | | | Extinct | 1.88 | | 1.64 | Yes | | Small |
|  | *Hydrictis gudho* | | | Extinct | 2 | | 1.88 | Yes | | Small |
|  | *Hydrictis sp.* | | | Extinct | 3.7 | | 3.5 | Yes | | Small |
|  | *Lutra hearsti* | | | Extinct | 3.4 | | 3.28 | Yes | | Small |
|  | *Lutrinae gen et sp nov* | | | Extinct | 2 | | 1.88 | Yes | | Small |
|  | *Mellivora sp.* | | | Extinct | 1.64 | | 1.39 | No | | Small |
|  | *Mellivora sp.* | | | Extinct | 2 | | 1.88 | No | | Small |
|  | *Mellivora sp.* | | | Extinct | 3.22 | | 3.18 | No | | Small |
|  | *Mellivora sp.* | | | Extinct | 3.85 | | 3.63 | No | | Small |
|  | *Mustelidae indet A* | | | Extinct | 3.22 | | 3.18 | No | | Small |
|  | *Mustelidae indet B* | | | Extinct | 3.22 | | 3.18 | No | | Small |
|  | *Mustelidae indet C* | | | Extinct | 3.18 | | 2.92 | No | | Small |
|  | *Mustelidae indet C* | | | Extinct | 3.22 | | 3.18 | No | | Small |
|  | *Mustelidae indet D* | | | Extinct | 3.18 | | 2.92 | No | | Small |
|  | *Prepoecilogale bolti* | | | Extinct | 2.66 | | 2.66 | No | | Small |
|  | *Prepoecilogale bolti* | | | Extinct | 3.85 | | 3.63 | No | | Small |
|  | *Torolutra cf. T. ougandensis* | | | Extinct | 3.07 | | 3 | Yes | | Small |
|  | *Torolutra cf. T. ougandensis* | | | Extinct | 3.36 | | 3.21 | Yes | | Small |
|  | *Torolutra cf. T. ougandensis* | | | Extinct | 4.23 | | 4.07 | Yes | | Small |
|  | *Torolutra sp.* | | | Extinct | 1.64 | | 1.39 | Yes | | Small |
|  | *Torolutra sp.* | | | Extinct | 1.88 | | 1.64 | Yes | | Small |
|  | *Torolutra sp.* | | | Extinct | 1.88 | | 1.65 | Yes | | Small |
|  | *Torolutra sp.* | | | Extinct | 2 | | 1.88 | Yes | | Small |
| Ursidae | *Cf. Agriotherium sp.* | | | Extinct | 3 | | 3 | No | | Large |
|  | *Cf. Agriotherium sp.* | | | Extinct | 3.18 | | 2.92 | No | | Large |
|  | *Cf. Agriotherium sp.* | | | Extinct | 3.22 | | 3.18 | No | | Large |
|  | *Cf. Agriotherium sp.* | | | Extinct | 3.36 | | 2.68 | No | | Large |
|  | *Cf. Agriotherium sp.* | | | Extinct | 3.36 | | 3.21 | No | | Large |
|  | *Cf. Agriotherium sp.* | | | Extinct | 4.35 | | 4.1 | No | | Large |
| Viverridae | *Civettictis aff. C. civetta* | | | Extinct | 2 | | 1.88 | No | | Small |
|  | *Civettictis aff. C. civetta* | | | Extinct | 2.52 | | 2.37 | No | | Small |
|  | *Civettictis aff. C. civetta* | | | Extinct | 3.21 | | 3 | No | | Small |
|  | *Civettictis aff. C. civetta* | | | Extinct | 3.5 | | 3.2 | No | | Small |
|  | *Civettictis aff. C. civetta* | | | Extinct | 3.7 | | 3.5 | No | | Small |
|  | *Genetta cf. G. servalina* | | | Extant | 3.7 | | 3.5 | No | | Small |
|  | *Genetta genetta/ maculata* | | | Extant | 1.64 | | 1.39 | No | | Small |
|  | *Genetta genetta/ maculata* | | | Extant | 2 | | 1.88 | No | | Small |
|  | *Genetta nyakitongwer* | | | Extinct | 1.88 | | 1.64 | No | | Small |
|  | *Peudocivetta ingens* | | | Extinct | 1.64 | | 1.39 | No | | Large |
|  | *Peudocivetta ingens* | | | Extinct | 1.7 | | 1.2 | No | | Large |
|  | *Peudocivetta ingens* | | | Extinct | 1.87 | | 1.7 | No | | Large |
|  | *Peudocivetta ingens* | | | Extinct | 1.88 | | 1.64 | No | | Large |
|  | *Peudocivetta ingens* | | | Extinct | 2 | | 1.88 | No | | Large |
|  | *Peudocivetta ingens* | | | Extinct | 2.33 | | 1.88 | No | | Large |
|  | *Peudocivetta ingens* | | | Extinct | 2.37 | | 2.33 | No | | Large |
|  | *Sahelictis cf. S.korei* | | | Extinct | 3.7 | | 3.5 | No | | Large |
|  | *Viverra leakeyi* | | | Extinct | 2.85 | | 2.52 | No | | Large |
|  | *Viverra leakeyi* | | | Extinct | 3.85 | | 3.63 | No | | Large |
|  | *Viverridae sp. A large* | | | Extinct | 3.22 | | 3.18 | No | | Small |
|  | *Viverridae sp. A large* | | | Extinct | 3.4 | | 3.28 | No | | Small |
|  | *Viverridae sp. A large* | | | Extinct | 3.9 | | 3.7 | No | | Small |
|  | *Viverridae sp. B small* | | | Extinct | 3.9 | | 3.7 | No | | Small |
|  | *Viverridae sp. C small* | | | Extinct | 3.9 | | 3.7 | No | | Small |
|  | *Viverridae sp. D* | | | Extinct | 3.36 | | 2.68 | No | | Small |
|  | *Viverridae sp. D* | | | Extinct | 3.5 | | 3.36 | No | | Small |
|  | *Viverridae sp. D* | | | Extinct | 4.35 | | 4.1 | No | | Small |
|  | *Viverridae sp. E* | | | Extinct | 3.36 | | 2.68 | No | | Small |
|  | *Viverridae sp. F* | | | Extinct | 2 | | 1.88 | No | | Small |
|  | *Viverridae sp. G* | | | Extinct | 1.88 | | 1.64 | No | | Small |

**Table S3. Estimated log marginal likelihoods (log marg. lik.) and log Bayes factors (log BF) quantifying the statistical fit of alternative extinction models.** Log marginal likelihoods and log BF were averaged across 100 randomized analyses with standard deviations given in parentheses. Log BF were computed between the best fitting model and all the others. Log BF greater than 2 indicate positive support in favor of the best model; log BF greater than 6 and 10 indicate strong and very strong support for the best model, respectively. When log BF is smaller, than 2 the simplest model (i.e. the model with fewer number of parameters) is preferred (preferred models are highlighted in bold).

|  |  | large | | large terrestrial | |
| --- | --- | --- | --- | --- | --- |
| Model | # parameters | log marg. lik. | log BF | log marg. lik. | log BF |
| Constant extinction | 2 | -277.56 (3.16) | 19.68 (3.21) | -245.29 (2.81) | 15.2 (2.31) |
| Extinction with 1 shift | 4 | -269.92 (2.73) | 4.38 (2.66) | -239.94 (2.41) | 4.5 (2.67) |
| Extinction with 2 shifts | 6 | -270.77 (3.03) | 6.1 (3.18) | -240.9 (2.73) | 6.41 (3.5) |
| Brain - exponential | 3 | -269.79 (2.03) | 4.12 (1.47) | -239.4 (1.92) | 3.41 (0.97) |
| **Brain - linear** | **3** | **-267.72 (2.16)** | **0 (0)** | **-237.69 (2.01)** | **0 (0)** |
| Brain - exponential, threshold | 4 | -270.85 (2.11) | 6.25 (1.62) | -240.47 (1.94) | 5.55 (1.28) |
| Brain - linear, threshold | 4 | -268.87 (2.15) | 2.29 (0.7) | -238.91 (1.99) | 2.43 (0.58) |
| Precipitation - exponential | 3 | -278.07 (2.76) | 20.68 (3.23) | -245.53 (2.2) | 15.68 (2.18) |
| Precipitation - linear | 3 | -277.72 (2.82) | 19.98 (3.43) | -244.9 (2.13) | 14.41 (2.51) |
| Temperature - exponential | 3 | -279.11 (3.19) | 22.77 (3.16) | -246.71 (2.9) | 18.03 (2.32) |
| Temperature - linear | 3 | -278.15 (3.21) | 20.85 (2.99) | -245.84 (2.95) | 16.3 (2.25) |
| Water deficit – exponential | 3 | -274.81 (2.34) | 14.12 (3.53) | -241.71 (1.5) | 8.24 (4.12) |
| Water deficit – linear | 3 | -275.66 (2.69) | 15.84 (3.81) | -242.07 (1.77) | 8.95 (4.33) |
| Forest cover - exponential | 3 | -267.73 (2.02) | 0.01 (2.88) | -238.07 (1.78) | 0.76 (2.8) |
| Forest cover - linear | 3 | -269.89 (2.09) | 4.33 (2.89) | -239.48 (1.83) | 3.58 (2.8) |
|  |  |  |  |  |  |
|  |  | small | | small terrestrial | |
| Model | # parameters | log marg. lik. | log BF | log marg. lik. | log BF |
| **Constant extinction** | **2** | **-360.88 (4.82)** | **0.25 (5.23)** | **-320.74 (5.07)** | **1.73 (6.31)** |
| Extinction with 1 shift | 4 | -361.8 (4.29) | 2.09 (3.57) | -321.54 (4.47) | 3.32 (4.8) |
| Extinction with 2 shifts | 6 | -360.75 (4.7) | 0 (0) | -319.88 (4.86) | 0 (0) |
| Brain - exponential | 3 | -362.1 (4.41) | 2.69 (4.87) | -321.69 (4.47) | 3.62 (5.74) |
| Brain - linear | 3 | -361.81 (4.42) | 2.11 (4.77) | -321.28 (4.34) | 2.81 (5.57) |
| Brain - exponential, threshold | 4 | -360.94 (4.72) | 0.38 (4.82) | -320.7 (4.87) | 1.65 (5.97) |
| Brain - linear, threshold | 4 | -360.86 (4.73) | 0.22 (4.38) | -320.62 (4.79) | 1.48 (5.56) |
| Precipitation - exponential | 3 | -362.75 (4.88) | 4 (4.51) | -322.6 (5.22) | 5.44 (6.03) |
| Precipitation - linear | 3 | -362.44 (4.78) | 3.37 (4.39) | -322.11 (5.1) | 4.46 (5.86) |
| Temperature - exponential | 3 | -362.69 (4.82) | 3.87 (5.24) | -322.39 (5.1) | 5.03 (6.23) |
| Temperature - linear | 3 | -362.16 (4.76) | 2.82 (5.1) | -321.61 (4.94) | 3.45 (5.73) |
| Water deficit – exponential | 3 | -360.95 (3.78) | 0.76 (5.85) | -321.36 (3.64) | 3.73 (8.63) |
| Water deficit – linear | 3 | -360.7 (3.77) | 0.26 (5.7) | -321.22 (3.7) | 3.45 (8.52) |
| Forest cover - exponential | 3 | -362.34 (4.38) | 3.17 (5.73) | -322.36 (3.87) | 4.87 (7.83) |
| Forest cover - linear | 3 | -362.69 (4.44) | 3.87 (5.92) | -322.75 (4.01) | 5.65 (7.84) |

**Table S4. Fraction of large North American carnivores by geological stage.** A few species had sizes spanning the threshold from Carbone et al. (1999) and these species are treated as 0.5 large species for calculation of this fraction. Presence/absence of each species by geological age and size can be found in Table S5.

| Epoch | Miocene | Pliocene | | Pleistocene | |
| --- | --- | --- | --- | --- | --- |
| Stage | Messinian | Zanclean | Piacenzian | Gelasian | Calabrian |
| Time span |  |  |  |  |  |
| Total carnivores | 64 | 58 | 58 | 46 | 53 |
| Large carnivores | 23.5 | 19.5 | 21.5 | 20 | 22.5 |
| Fraction large | 37% | 34% | 37% | 43% | 42% |

**Table S5. Size and presence by geological stage for North American carnivores.** All records are based on data from Faurby et al. (2019).

| Family | Species | Calabrian | Gelasian | Piacenzian | Zanclean | Messinian | Size |
| --- | --- | --- | --- | --- | --- | --- | --- |
| Ailuridae | *Parailurus NorthAmerica* | Absent | Absent | Present | Present | Absent | Small |
| Ailuridae | *Pristinailurus bristoli* | Absent | Absent | Absent | Present | Present | Small |
| Barbourofelidae | *Albanosmilus whitfordi* | Absent | Absent | Absent | Absent | Present | Large |
| Barbourofelidae | *Barbourofelis fricki* | Absent | Absent | Absent | Absent | Present | Large |
| Canidae | *Aelurodon taxoides* | Absent | Absent | Absent | Absent | Present | Large |
| Canidae | *Borophagus diversidens* | Present | Present | Present | Present | Present | Large |
| Canidae | *Borophagus dudleyi* | Absent | Absent | Absent | Present | Present | Large |
| Canidae | *Borophagus hilli* | Absent | Absent | Present | Present | Present | Large |
| Canidae | *Borophagus littoralis* | Absent | Absent | Absent | Absent | Present | Overlapping between large and small |
| Canidae | *Borophagus orc* | Absent | Absent | Absent | Present | Present | Small |
| Canidae | *Borophagus parvus* | Absent | Absent | Absent | Absent | Present | Small |
| Canidae | *Borophagus pugnator* | Absent | Absent | Absent | Absent | Present | Large |
| Canidae | *Borophagus secundus* | Absent | Absent | Present | Present | Present | Overlapping between large and small |
| Canidae | *Canis armbrusteri* | Present | Present | Present | Absent | Absent | Small |
| Canidae | *Canis dirus* | Present | Absent | Absent | Absent | Absent | Large |
| Canidae | *Canis edwardii* | Present | Present | Present | Present | Present | Large |
| Canidae | *Canis ferox* | Absent | Absent | Present | Present | Present | Small |
| Canidae | *Canis latrans* | Present | Present | Present | Absent | Absent | Small |
| Canidae | *Canis lepophagus* | Present | Present | Present | Present | Present | Small |
| Canidae | *Canis lupus* | Present | Absent | Absent | Absent | Absent | Large |
| Canidae | *Canis rufus* | Present | Present | Absent | Absent | Absent | Large |
| Canidae | *Canis thooides* | Absent | Present | Present | Absent | Absent | Small |
| Canidae | *Carpocyon limosus* | Absent | Absent | Absent | Absent | Present | Small |
| Canidae | *Cerdocyon avius* | Absent | Absent | Absent | Present | Absent | Small |
| Canidae | *Cerdocyon texanus* | Absent | Absent | Present | Present | Present | Small |
| Canidae | *Chrysocyon nearcticus* | Absent | Present | Present | Present | Absent | Small |
| Canidae | *Epicyon haydeni* | Absent | Absent | Absent | Absent | Present | Large |
| Canidae | *Epicyon saevus* | Absent | Absent | Absent | Absent | Present | Large |
| Canidae | *Eucyon davisi* | Absent | Absent | Absent | Present | Present | Small |
| Canidae | *Metalopex bakeri* | Absent | Absent | Absent | Absent | Present | Small |
| Canidae | *Metalopex merriami* | Absent | Absent | Absent | Present | Present | Small |
| Canidae | *Theriodictis floriadanus* | Absent | Present | Present | Present | Absent | Small |
| Canidae | *Urocyon cinereoargenteus* | Present | Present | Present | Absent | Absent | Small |
| Canidae | *Urocyon citrinus* | Absent | Present | Present | Absent | Absent | Small |
| Canidae | *Urocyon galushai* | Present | Present | Present | Present | Absent | Small |
| Canidae | *Urocyon progressus* | Absent | Absent | Present | Present | Absent | Small |
| Canidae | *Vulpes kernensis* | Absent | Absent | Absent | Absent | Present | Small |
| Canidae | *Vulpes lagopus* | Present | Absent | Absent | Absent | Absent | Small |
| Canidae | *Vulpes stenognathus* | Present | Present | Present | Present | Present | Small |
| Canidae | *Vulpes velox* | Present | Present | Present | Present | Absent | Small |
| Canidae | *Vulpes vulpes* | Present | Absent | Absent | Absent | Absent | Small |
| Felidae | *Adelphailurus kansensis* | Absent | Absent | Absent | Absent | Present | Large |
| Felidae | *Amphimachairodus coloradensis* | Absent | Absent | Absent | Present | Present | Large |
| Felidae | *Dinofelis palaeoonca* | Absent | Present | Present | Absent | Absent | Large |
| Felidae | *Homotherium ischyrus* | Absent | Absent | Absent | Present | Absent | Large |
| Felidae | *Homotherium serum* | Present | Present | Present | Present | Absent | Large |
| Felidae | *Leopardus amnicola* | Present | Absent | Absent | Absent | Absent | Small |
| Felidae | *Lynx canadensis* | Present | Absent | Absent | Absent | Absent | Small |
| Felidae | *Lynx issiodorensis* | Absent | Present | Absent | Absent | Absent | Large |
| Felidae | *Lynx proterolyncis* | Absent | Absent | Absent | Absent | Present | Small |
| Felidae | *Lynx rufus* | Present | Present | Present | Absent | Absent | Small |
| Felidae | *Machairodus tanneri* | Absent | Absent | Absent | Present | Present | Large |
| Felidae | *Megantereon hesperus* | Present | Present | Present | Present | Absent | Large |
| Felidae | *Miracinonyx inexpectatus* | Present | Present | Present | Absent | Absent | Large |
| Felidae | *Miracinonyx trumani* | Present | Present | Present | Present | Absent | Large |
| Felidae | *Nimravides catacopsis* | Absent | Absent | Absent | Present | Present | Large |
| Felidae | *Panthera atrox* | Present | Absent | Absent | Absent | Absent | Large |
| Felidae | *Panthera onca* | Present | Present | Present | Absent | Absent | Large |
| Felidae | *Puma concolor* | Present | Present | Present | Absent | Absent | Large |
| Felidae | *Puma lacustris* | Absent | Present | Present | Present | Present | Large |
| Felidae | *Rhizosmilodon fiteae* | Absent | Absent | Absent | Absent | Present | Large |
| Felidae | *Smilodon fatalis* | Present | Absent | Absent | Absent | Absent | Large |
| Felidae | *Smilodon gracilis* | Present | Present | Present | Present | Absent | Large |
| Felidae | *Xenosmilus hodsonae* | Present | Present | Absent | Absent | Absent | Large |
| Hyanidae | *Chasmaporthetes ossifragus* | Present | Present | Present | Present | Absent | Large |
| Mephetidae | *Brachyopsigale dubius* | Absent | Absent | Present | Present | Present | Small |
| Mephetidae | *Brachyprotoma obtusata* | Present | Absent | Absent | Absent | Absent | Small |
| Mephetidae | *Buisnictis breviramus* | Absent | Present | Present | Present | Absent | Small |
| Mephetidae | *Buisnictis burrowsi* | Absent | Absent | Present | Present | Absent | Small |
| Mephetidae | *Buisnictis meadensis* | Absent | Absent | Present | Present | Present | Small |
| Mephetidae | *Buisnictis metabatos* | Absent | Absent | Absent | Present | Absent | Small |
| Mephetidae | *Martinogale alveodens* | Absent | Absent | Absent | Absent | Present | Small |
| Mephetidae | *Mephitis mephitis* | Present | Present | Present | Present | Absent | Small |
| Mephetidae | *Pliogale furlongi* | Absent | Absent | Absent | Absent | Present | Small |
| Mephetidae | *Spilogale microdens* | Absent | Absent | Present | Present | Absent | Small |
| Mephetidae | *Spilogale putorius* | Present | Present | Present | Absent | Absent | Small |
| Mephetidae | *Spilogale rexroadi* | Absent | Present | Present | Present | Absent | Small |
| Mustelidae | *Arctomeles dimolodontus* | Absent | Absent | Absent | Present | Present | Small |
| Mustelidae | *Arctomeles sotnikovae* | Absent | Absent | Absent | Present | Absent | Small |
| Mustelidae | *Cernictis hesperus* | Absent | Absent | Absent | Absent | Present | Small |
| Mustelidae | *Cernictis repenningi* | Absent | Absent | Absent | Absent | Present | Small |
| Mustelidae | *Chamitaxus avitus* | Absent | Absent | Absent | Absent | Present | Small |
| Mustelidae | *Enhydra lutris* | Present | Absent | Absent | Absent | Absent | Large |
| Mustelidae | *Enhydritherium terraenovae* | Absent | Absent | Present | Present | Present | Large |
| Mustelidae | *Eomellivora NorthAmerica* | Absent | Absent | Absent | Absent | Present | Large |
| Mustelidae | *Ferinestrix vorax* | Absent | Absent | Absent | Present | Absent | Small |
| Mustelidae | *Gulo gulo* | Present | Absent | Absent | Absent | Absent | Overlapping between large and small |
| Mustelidae | *Leptarctus desuii* | Absent | Absent | Absent | Absent | Present | Small |
| Mustelidae | *Leptarctus supremus* | Absent | Absent | Absent | Absent | Present | Small |
| Mustelidae | *Leptarctus woodburnei* | Absent | Absent | Absent | Absent | Present | Small |
| Mustelidae | *Lontra canadensis* | Present | Present | Absent | Absent | Absent | Small |
| Mustelidae | *Lontra weiri* | Absent | Absent | Absent | Present | Absent | Small |
| Mustelidae | *Lutravus halli* | Absent | Absent | Absent | Absent | Present | Small |
| Mustelidae | *Martes diluviana* | Present | Absent | Absent | Absent | Absent | Small |
| Mustelidae | *Martes oregonensis* | Absent | Absent | Absent | Absent | Present | Small |
| Mustelidae | *Martes stirtoni* | Absent | Absent | Absent | Absent | Present | Small |
| Mustelidae | *Mionictis angustidens* | Absent | Absent | Absent | Absent | Present | Small |
| Mustelidae | *Mustela erminea* | Present | Absent | Absent | Absent | Absent | Small |
| Mustelidae | *Mustela frenata* | Present | Present | Present | Absent | Absent | Small |
| Mustelidae | *Mustela jacksoni* | Present | Absent | Absent | Absent | Absent | Small |
| Mustelidae | *Mustela meltoni* | Absent | Absent | Present | Present | Absent | Small |
| Mustelidae | *Mustela nigripes* | Present | Absent | Absent | Absent | Absent | Small |
| Mustelidae | *Mustela nivalis* | Present | Absent | Absent | Absent | Absent | Small |
| Mustelidae | *Mustela rexroadensis* | Absent | Present | Present | Present | Present | Small |
| Mustelidae | *Neovison vison* | Present | Absent | Absent | Absent | Absent | Small |
| Mustelidae | *Plesiogulo lindsayi* | Absent | Absent | Absent | Absent | Present | Large |
| Mustelidae | *Plesiogulo marshalli* | Absent | Absent | Absent | Absent | Present | Overlapping between large and small |
| Mustelidae | *Pliotaxidea garberi* | Absent | Absent | Absent | Absent | Present | Small |
| Mustelidae | *Pliotaxidea nevadensis* | Absent | Absent | Absent | Absent | Present | Small |
| Mustelidae | *Satherium piscinarium* | Present | Present | Present | Present | Present | Small |
| Mustelidae | *Sminthosinis bowleri* | Absent | Absent | Present | Present | Present | Small |
| Mustelidae | *Taxidea mexicana* | Absent | Absent | Absent | Absent | Present | Small |
| Mustelidae | *Taxidea taxus* | Present | Present | Present | Present | Absent | Small |
| Mustelidae | *Tisisthenes parvus* | Present | Absent | Absent | Absent | Absent | Small |
| Mustelidae | *Trigonictis cookii* | Present | Present | Present | Present | Absent | Small |
| Mustelidae | *Trigonictis macrodon* | Present | Present | Present | Present | Absent | Small |
| Procyonidae | *Arctonasua eurybates* | Absent | Absent | Absent | Absent | Present | Small |
| Procyonidae | *Arctonasua fricki* | Absent | Absent | Absent | Absent | Present | Small |
| Procyonidae | *Bassariscus casei* | Present | Present | Present | Present | Absent | Small |
| Procyonidae | *Bassariscus ogallalae* | Absent | Absent | Absent | Absent | Present | Small |
| Procyonidae | *Nasua pronarica* | Absent | Absent | Present | Present | Absent | Small |
| Procyonidae | *Probassariscus matthewi* | Absent | Absent | Absent | Absent | Present | Small |
| Procyonidae | *Procyon lotor* | Present | Present | Present | Present | Present | Small |
| Procyonidae | *Procyon rexroadensis* | Absent | Present | Present | Present | Absent | Small |
| Ursidae | *Agriotherium schneideri* | Absent | Absent | Present | Present | Present | Large |
| Ursidae | *Arctodus pristinus* | Present | Present | Present | Absent | Absent | Large |
| Ursidae | *Arctodus simus* | Present | Present | Absent | Absent | Absent | Large |
| Ursidae | *Arctotherium NorthAmerica* | Absent | Absent | Present | Absent | Absent | Large |
| Ursidae | *Indarctos oregonensis* | Absent | Absent | Absent | Absent | Present | Large |
| Ursidae | *Plionarctos edensis* | Absent | Present | Present | Present | Present | Large |
| Ursidae | *Plionarctos harroldorum* | Absent | Absent | Present | Present | Absent | Large |
| Ursidae | *Tremarctos floridanus* | Present | Present | Present | Absent | Absent | Large |
| Ursidae | *Ursus americanus* | Present | Present | Present | Present | Absent | Large |
| Ursidae | *Ursus arctos* | Present | Absent | Absent | Absent | Absent | Large |

**Table S6. Parameter estimates under 15 extinction models in large carnivores.** The parameters are summarized as mean and 95% highest posterior density intervals (HPD) after combining the output of 100 replicated analyses (see Methods). Models with rate shifts include extinction rates between shifts (mu_0, mu_1, ...) and times of rate shift (shift_time) in Ma. Models in which extinction rate is a function of a time-continuous variable (temperature, precipitations, brain size) include a baseline extinction rate (mu_0) and a correlation parameter (alpha and g for exponential and linear models, respectively. Models with correlations to brain size with a threshold, additionally include the time (in Ma) after which the brain size is estimated to affect extinction.

|  | | | |  | | | Large | | | | | |  | | Large terrestrial | | | | |
| --- | --- | --- | --- | --- | --- | --- | --- | --- | --- | --- | --- | --- | --- | --- | --- | --- | --- | --- | --- |
| Model | | | | parameter | | | mean | | | HPD | | |  | | mean | | | HPD_min | |
| Constant extinction | | | | mu_0 | | | 0.552 | | | 0.366, 0.753 | | |  | | 0.552 | | | 0.362, 0.760 | |
| Extinction with 1 shift | | | | mu_0 | | | 0.271 | | | 0.109, 0.429 | | |  | | 0.278 | | | 0.117, 0.482 | |
|  | | | | mu_1 | | | 1.616 | | | 0.851, 2.521 | | |  | | 1.519 | | | 0.710, 2.409 | |
|  | | | | shift_time_1 | | | 1.854 | | | 1.620, 2.296 | | |  | | 1.844 | | | 1.486, 2.411 | |
| Extinction with 2 shifts | | | | mu_0 | | | 0.257 | | | 0.000, 0.432 | | |  | | 0.259 | | | 0.001, 0.456 | |
|  | | | | mu_1 | | | 1.530 | | | 0.104, 3.404 | | |  | | 1.320 | | | 0.001, 2.907 | |
|  | | | | mu_2 | | | 1.147 | | | 0.001, 2.237 | | |  | | 1.148 | | | 0.001, 2.262 | |
|  | | | | shift_time_1 | | | 2.333 | | | 1.613, 3.769 | | |  | | 2.415 | | | 1.514, 3.817 | |
|  | | | | shift_time_2 | | | 1.239 | | | 0.347, 2.067 | | |  | | 1.247 | | | 0.286, 1.998 | |
| Brain volume - exponential | | | | mu_0 | | | 0.53 | | | 0.339, 0.733 | | |  | | 0.532 | | | 0.327, 0.749 | |
|  | | | | alpha | | | 2.862 | | | 1.604, 4.231 | | |  | | 2.705 | | | 1.291, 4.094 | |
| Brain volume - linear | | | | mu_0 | | | 0.702 | | | 0.440, 0.927 | | |  | | 0.700 | | | 0.453, 0.971 | |
|  | | | | g | | | 2.882 | | | 2.002, 3.699 | | |  | | 2.747 | | | 1.877, 3.541 | |
| Precipitation - exponential | | | | mu_0 | | | 0.542 | | | 0.366, 0.739 | | |  | | 0.549 | | | 0.340, 0.746 | |
|  | | | | alpha | | | -1.023 | | | -2.405, 0.416 | | |  | | -1.143 | | | -2.583, 0.371 | |
| Precipitation – linear | | | | mu_0 | | | 0.576 | | | 0.379, 0.768 | | |  | | 0.595 | | | 0.388, 0.819 | |
|  | | | | g | | | -1.322 | | | -3.046, 0.190 | | |  | | -1.590 | | | -3.576, 0.210 | |
| Temperature - exponential | | | | mu_0 | | | 0.508 | | | 0.312, 0.716 | | |  | | 0.511 | | | 0.310, 0.732 | |
|  | | | | alpha | | | -0.549 | | | -2.092, 1.079 | | |  | | -0.618 | | | -2.183, 0.95 | |
| Temperature- linear | | | | mu_0 | | | 0.504 | | | 0.299, 0.697 | | |  | | 0.492 | | | 0.311, 0.702 | |
|  | | | | g | | | -1.350 | | | -3.842, 0.988 | | |  | | -1.350 | | | -3.710, 0.928 | |
| Water deficit - exponential | | | | mu_0 | | | 0.071 | | | 0.047, 0.097 | | |  | | 0.070 | | | 0.046, 0.097 | |
|  | | | | alpha | | | 1.852 | | | 0.652, 3.072 | | |  | | 1.985 | | | 0.712, 3.250 | |
| Water deficit - linear | | | | mu_0 | | | 0.076 | | | 0.047, 0.108 | | |  | | 0.080 | | | 0.048, 0.113 | |
|  | | | | g | | | 1.283 | | | 0.552, 1.901 | | |  | | 1.458 | | | 0.652, 2.114 | |
| Forest cover - exponential | | | | mu_0 | | | 0.481 | | | 0.280, 0.681 | | |  | | 0.476 | | | 0.279, 0.686 | |
|  | | | | alpha | | | -2.455 | | | -3.800, -1.421 | | |  | | -2.287 | | | -3.504, -1.157 | |
| Forest cover - linear | | | | mu_0 | | | 0.672 | | | 0.434, 0.899 | | |  | | 0.649 | | | 0.412, 0.882 | |
|  | | | | g | | | -1.575 | | | -2.026, -1.062 | | |  | | -1.532 | | | -2.021, -0.982 | |
| Brain volume threshold - exponential | | | | mu_0 | | | 0.601 | | | 0.301, 0.747 | | |  | | 0.588 | | | 0.298, 0.778 | |
|  | | | | alpha | | | 2.735 | | | 1.204, 4.498 | | |  | | 2.593 | | | 1.081, 4.582 | |
|  | | | | threshold | | | 3.490 | | | 2.234, 4.382 | | |  | | 3.419 | | | 2.114, 4.366 | |
| Brain volume threshold - linear | | | | mu_0 | | | 0.694 | | | 0.434, 0.961 | | |  | | 0.671 | | | 0.408, 0.941 | |
|  | | | | g | | | 3.235 | | | 2.175, 4.994 | | |  | | 3.017 | | | 1.705, 4.832 | |
|  | | | | threshold | | | 3.478 | | | 2.423, 4.349 | | |  | | 3.480 | | | 2.397, 4.348 | |
| **Table S7. Preservation rates estimated in different time bins.** Preservation rates indicate the expected number of fossil occurrences per sampled lineage per million years and were estimated using PyRate. To reflect the sampling distribution of the fossil occurrences we binned the preservation process in three time-windows with rate shifts at 4.36 and 0.99 Ma (see Methods). The table provides posterior estimates of the preservation rate prior to 4.36 Ma (q_0), between 4.36 and 0.99 Ma (q_1), and after 0.99 Ma (q_2). Rates in the first and third intervals are lowest, reflecting the empirical distribution of sampled fossil ages. The estimates summarise 100 randomised analyses (see Methods). | | | | | | | | | | | | | | | | | | |  |
|  | large | | | | large terrestrial | | | | small | | | | | small terrestrial | | | | |  |
|  | q_0 | q_1 | q_2 | | q_0 | q_1 | | q_2 | q_0 | | q_1 | q_2 | | q_0 | | q_1 | q_2 | |  |
| mean | 2.27 | 2.88 | 1.87 | | 2.41 | 3.04 | | 1.97 | 0.32 | | 1.17 | 0.19 | | 0.35 | | 1.03 | 0.17 | |  |
| median | 2.40 | 2.88 | 1.82 | | 2.44 | 3.03 | | 1.96 | 0.22 | | 1.17 | 0.13 | | 0.23 | | 1.03 | 0.13 | |  |
| 95% HPD lower | 0.04 | 2.27 | 0.12 | | 0.07 | 2.40 | | 0.24 | 0.00 | | 0.77 | 0.00 | | 0.00 | | 0.76 | 0.00 | |  |
| 95% HPD upper | 3.61 | 3.47 | 4.25 | | 3.78 | 3.81 | | 4.06 | 0.81 | | 1.50 | 0.46 | | 0.72 | | 1.39 | 0.44 | |  |

**Table S8. Parameter estimates under 15 extinction models in small carnivores.** The parameters are summarized as mean and 95% highest posterior density intervals (HPD) after combining the output of 100 replicated analyses (see Methods). The parameters are as in Table S5.

|  |  | Small | |  | Small terrestrial | |
| --- | --- | --- | --- | --- | --- | --- |
| Model | parameter | mean | HPD_min |  | mean | HPD_min |
| Constant extinction | mu_0 | 0.717 | 0.525, 0.941 |  | 0.637 | 0.444, 0.846 |
| Extinction with 1 shift | mu_0 | 0.685 | 0.256, 1.029 |  | 0.557 | 0.195, 1.027 |
|  | mu_1 | 1.056 | 0.013, 1.787 |  | 1.007 | 0.002, 1.729 |
|  | shift_time_1 | 1.85 | 0.476, 3.724 |  | 1.87 | 0.647, 3.778 |
| Extinction with 2 shifts | mu_0 | 0.784 | 0.086, 1.607 |  | 0.576 | 0.077, 1.153 |
|  | mu_1 | 1.525 | 0.002, 3.089 |  | 1.858 | 0.022, 3.806 |
|  | mu_2 | 0.553 | 0, 1.568 |  | 0.452 | 0, 1.327 |
|  | shift_time_1 | 2.16 | 1.435, 4.059 |  | 2.025 | 1.408, 3.8 |
|  | shift_time_2 | 1.164 | 0.345, 2.209 |  | 1.136 | 0.294, 2.099 |
| Brain volume - exponential | mu_0 | 0.703 | 0.511, 0.92 |  | 0.626 | 0.419, 0.832 |
|  | alpha | 0.714 | -0.405, 1.933 |  | 0.858 | -0.497, 2.186 |
| Brain volume - linear | mu_0 | 0.724 | 0.519, 0.938 |  | 0.645 | 0.436, 0.854 |
|  | g | 0.828 | -0.392, 2.013 |  | 0.991 | -0.282, 2.383 |
| Precipitation - exponential | mu_0 | 0.716 | 0.52, 0.94 |  | 0.63 | 0.434, 0.843 |
|  | alpha | 0.058 | -1.287, 1.436 |  | 0.1 | -1.618, 1.549 |
| Precipitation - linear | mu_0 | 0.719 | 0.521, 0.949 |  | 0.638 | 0.434, 0.85 |
|  | g | -0.237 | -1.972, 1.237 |  | -0.229 | -2.321, 1.465 |
| Temperature - exponential | mu_0 | 0.695 | 0.489, 0.944 |  | 0.601 | 0.383, 0.83 |
|  | alpha | -0.242 | -1.705, 1.074 |  | -0.416 | -2.011, 1.047 |
| Temperature- linear | mu_0 | 0.696 | 0.476, 0.95 |  | 0.593 | 0.352, 0.809 |
|  | g | -0.557 | -2.446, 1.183 |  | -0.991 | -3.167, 1.038 |
| Water deficit - exponential | mu_0 | 0.082 | 0.057, 0.107 |  | 0.073 | 0.049, 0.097 |
|  | alpha | 0.971 | -0.092, 2.031 |  | 1.038 | -0.082, 2.127 |
| Water deficit - linear | mu_0 | 0.088 | 0.058, 0.119 |  | 0.078 | 0.050, 0.107 |
|  | g | 0.952 | 0.060, 1.775 |  | 0.941 | 0.050, 1.725 |
| Forest cover - exponential | mu_0 | 0.709 | 0.503, 0.925 |  | 0.618 | 0.418, 0.82 |
|  | alpha | -0.636 | -1.413, 0.147 |  | -0.78 | -1.749, 0.099 |
| Forest cover - linear | mu_0 | 0.732 | 0.519, 0.948 |  | 0.649 | 0.446, 0.858 |
|  | g | -0.514 | -1.148, 0.169 |  | -0.596 | -1.294, 0.166 |
| Brain volume threshold - exponential | mu_0 | 2.767 | 0.1, 7.86 |  | 2.215 | 0.048, 6.559 |
|  | alpha | -1.976 | -4.998, 1.609 |  | -1.607 | -4.997, 1.967 |
|  | threshold | 1.391 | 0.051, 3.796 |  | 1.561 | 0.058, 3.976 |
| Brain volume threshold - linear | mu_0 | 3.385 | 0.146, 13.084 |  | 3.028 | 0.142, 12.278 |
|  | g | -0.185 | -2.512, 3.731 |  | -0.008 | -2.549, 3.857 |
|  | threshold | 1.66 | 0.09, 3.979 |  | 1.761 | 0.07, 3.997 |

**Figure S1. Diversity trajectory of large carnivores broken down by sub-guild.** The average diversity across the 100 replicates of large carnivores (>21 kg) within each of six taxonomical/ ecological sub-guilds. Three of these are extant in the area and are shown in bolder lines (Conical toothed cats, hyenas and dogs) and three are regional (giant omnivores) or globally extinct (giant otters and saber-toothed cats) and are shown in thinner lines.
